# Fishing, predation, and temperature drive herring decline in a large marine ecosystem

**DOI:** 10.1002/ece3.8411

**Published:** 2021-12-14

**Authors:** Daniel G. Boyce, Brian Petrie, Kenneth T. Frank

**Affiliations:** ^1^ Ocean Sciences Division Bedford Institute of Oceanography Dartmouth NS Canada; ^2^ Biology Department Dalhousie University Halifax NS Canada

**Keywords:** Atlantic herring, climate change, ecosystem, ecosystem‐based, egg predation, exploitation, fisheries, forage, herring

## Abstract

Since 1960, landings of Atlantic herring have been the greatest of any marine species in Canada, surpassing Atlantic cod and accounting for 24% of the total seafood harvested in Atlantic Canada. The Scotian Shelf‐Bay of Fundy herring fisheries (NAFO Division 4VWX) is among Canada's oldest and drives this productivity, accounting for up to 75% of the total herring catch in some years. The stocks’ productivity and overall health have declined since 1965. Despite management measures to promote recovery implemented since 2003, biomass remains low and is declining. The factors that drive the productivity of 4VWX herring are primarily unresolved, likely impeding the effectiveness of management actions on this stock. We evaluated potential drivers of herring variability by analyzing 52 time‐series that describe the temporal and spatial evolution of the 4VWX herring population and the physical, ecological, and anthropogenic factors that could affect them using structural equation models. Variation in herring biomass was best accounted for by the exploitation rate's negative effect and the geographic distribution of fishing and recruitment. Thermal phenology and temperature adversely and egg predation positively impacted the early life stage mortality rate and, ultimately, adult biomass. These findings are broadly relevant to fisheries management, but particularly for 4VWX herring, where the current management approach does not consider their early life stage dynamics or assess them within the ecosystem or climate change contexts.

## Graphical abstract

The productivity and overall health of Scotian Shelf‐Bay of Fundy herring (NAFO Division 4VWX) have declined since 1965 and have failed to improve despite recovery measures in place since 2003. We evaluated potential drivers of herring decline by analyzing 52‐time series that describe the temporal and spatial evolution of the 4VWX herring population and the physical, ecological, and anthropogenic factors that could affect them using structural equation models. Variation in herring biomass was best explained by the adverse effect of exploitation rate and positive impact of the geographic distribution of fishing and recruitment, while thermal phenology, egg predation, and temperature impacted the early life stage mortality rate and, ultimately, adults’ biomass.

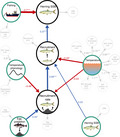

## 1 INTRODUCTION

The heavy exploitation combined with the socio‐economic and ecological importance of forage fish globally has raised concerns about their conservation status (FAO, 2014). Hypotheses to explain forage fish population fluctuations and collapses have been diverse: heavy exploitation (Essington et al., 2015), high, early life stage mortality (Fassler et al., 2011), intrinsic density‐dependent population regulation (Myers & Barrowman, 1996), predation (Kotterba et al., 2017; Richardson et al., 2011), and environmental variability (Brosset et al., 2018; Trochta et al., 2020). Traditional management approaches focus heavily on the role of exploitation in regulating recruitment and adult biomass and largely overlook the importance of ecosystem or climate factors. However, some forage fish stock collapses have no apparent explanation or have occurred naturally (Peck et al., 2014). The inability of exploitation alone to explain forage fish population collapses (McClatchie et al., 2017; Peck et al., 2014) or delayed recoveries (Essington et al., 2015) suggests that additional factors are critical in accounting for population variability.

**FIGURE 1:**
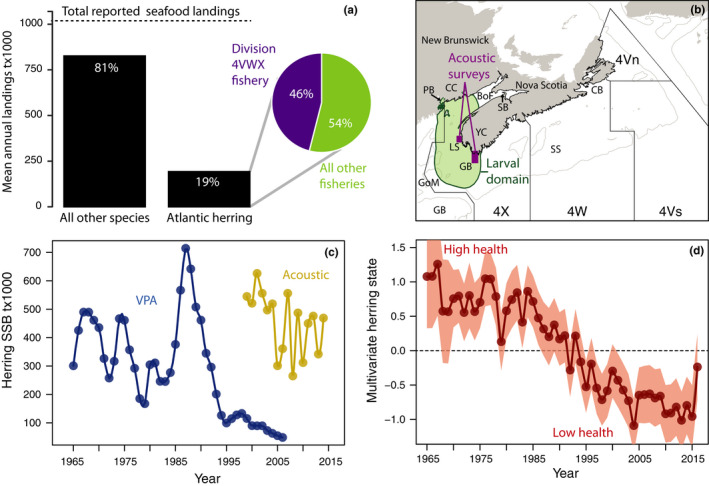
Overview of the Atlantic herring fishery in NAFO division 4VWX. (a) Average annual herring landings in Division 4VWX (purple), compared with those of all other herring fisheries (green) or other marine fisheries in Canada between 1990 and 2018. The dashed horizontal line depicts the average of all seafood landed in Canada annually. (b) Spatial distribution of acoustic biomass (purple; 1999–2019) and larval (green; 1975–1998) surveys used to calculate herring SSB. (c) time‐series of herring SSB estimated from a population model (VPA; blue) or acoustic survey estimates (yellow). (d) Multivariate time‐series of standardized herring population health derived from 16 state indicators, where high values denote positive population health and low negative (Boyce et al., 2019). The red shading depicts the 95% CI about the averages. Abbreviated place names in (b): CC = Charlotte County; YC = Yarmouth County; PB = Passamaquoddy Bay; BoF = Bay of Fundy; LS = Lurcher Shoal; GB = German Bank; GoM = Gulf of Maine; SS = Scotian Shelf; CB = Chedabucto Bay; SB = Scotts Bay; GB = Georges Bank

Atlantic herring (*Clupea harengus*), one of Canada's oldest and largest fisheries, has been experiencing a long‐term decline over the broad Scotian Shelf and Bay of Fundy region (NAFO Division 4VWX; Figure 1a,b). It accounted for 19% of Canada's total seafood landings between 1990 and 2018, the largest of any species (Figure 1a). However, the productivity and population health of 4VWX herring, defined by several metrics, have declined since 1965 and rapidly throughout the 1980s, reaching historically low biomass levels after 2005 (Boyce et al., 2019); Figure 1c,d). Based on a recent review of 64 herring stocks by Trochta et al. (2020), the decline in 4VWX herring stock status is exceptional: it has been in a collapsed state for 26 years (1994–2019), far longer than the average collapse duration of 11 years. The causes of the long‐term decline in herring state (Figure 1d) and the failure of the stock to respond to reduced exploitation are unknown, impairing management and conservation efforts. The cultural, economic, and ecological importance of herring within this region provides a strong incentive to understand the factors which underlie its declining status.

The 4VWX herring stock undergoes a complex seasonal cycle of spawning, overwintering, and feeding that involves separate geographic domains and differential mixing with neighboring populations. Mark and recapture studies indicate that adult herring (>2–3 years old) spawn near German Bank/Lurcher Shoals in August–November, overwinter ~700 km to the northeast in Chedabucto Bay in January–March, and feed on the Scotian Shelf and Bay of Fundy in April–July (Stobo & Fowler, 2009). Upon hatching and before metamorphosis, larval herring remain within a well‐defined, tidally‐mixed “larval retention area” (LRA) off SWNS (Iles & Sinclair, 1982; Stephenson et al., 2015). Juvenile herring join a localized, nearshore migrant juvenile community near Passamaquoddy Bay along with juveniles from the Gulf of Maine and Georges Bank.

The 4VWX herring fishery targets spawning and nonspawning adult herring and juveniles (sardines) using multiple fishing gears, primarily purse seines but also weirs and gillnets. From 1985 to 2014, 69% of the effort was directed mainly to harvest roe during the spawning season (Singh et al., 2016). Unlike some roe fisheries, such as Pacific herring, which harvest roe after it is shed from females, adult herring are captured and then processed for their roe. The fishery is managed as four components, with the dominant Southwest Nova Scotia/Bay of Fundy (SWNS/BoF) component accounting for up to 93% of all landings in some years (DFO, 2015). Of these, the SWNS spawning component has directly accounted for a far greater fraction (44%) of the total herring landed annually in Division 4VWX (1985–2014) relative to the upper BoF (10%). Accordingly, due to its greater productivity and availability of datasets, our evaluation and discussion of 4VWX herring focused on the SWNS spawning herring complex.

Here, we evaluated how the physical, ecological, and anthropogenic conditions individually and synergistically affect the population dynamics of 4VWX herring across different life stages. To achieve this goal, we assembled time‐series of the herring stock's intrinsic population characteristics across different life stages and coincident series of climate, weather, chemistry, biology, exploitation, and ecological dynamics (predation, prey availability, competition) that could plausibly influence population productivity. We used multivariate statistical approaches, including multi‐model inference and structural equation models, to explore hypotheses for how these factors interact and affect herring population variability. We interpret our findings in the context of forage fish dynamics and suggest ways to improve forage fish management, particularly 4VWX herring.

## 2 MATERIALS AND METHODS

### 2.1 Overview of approach

The herring stock has a long, detailed assessment history based on surveys designed to collect larval and adult herring and collation of landings data from each commercial fisheries sector (*e*.*g*. DFO, 2015). Various research initiatives have generated knowledge about distribution, growth, and maturity at different life stages. We used biologically relevant indicators from several data sources to define the spatial and temporal variability required to explore the drivers of herring variability across the different life stages. Before testing hypotheses, individual time‐series were standardized to common spatial and temporal resolutions and measurement units for 1975–2005. The preprocessing steps were (1) defining the spatial, seasonal, and temporal domains, (2) data acquisition and calculation of the indicator time‐series, (3) imputation, normalization, and standardization of time‐series, and (4) statistical analyses.

#### 2.1.1 Defining the spatial, seasonal, and temporal domains

The geographic boundaries used to define larval and adult herring habitat were identified by Boyce et al. (2019). The habitat domain of 4VWX larval herring (the larval retention area; LRA, see Figure 1b) was estimated using field observations of their abundance from the Bay of Fundy larval herring survey and particle transport simulations from WebDrogue (Hannah et al., 2001). The spatial domain of adult herring in 4VWX was previously defined by Boyce et al. (2019) using survey observations, tagging studies, and landings statistics and includes most of the 4VWX domain. Herring in this area could originate from other spawning complexes located on the offshore banks or in the United States and represent an unknown mixture of stock sub‐components.

The biophysical data series that potentially govern 4VWX herring productivity at early life stages were restricted to the LRA and the July 15–October 31 spawning window. The biophysical data series used to explore adult herring dynamics were restricted to the adult spatial domain. We present available data for the 1970–2010 period; due to limitations of some of the series, our statistical modeling analyses concentrate on the 1975–2005 period. Much of the variation in herring population state over the past 50 years occurred during these periods (Boyce et al., 2019; DFO, 2018; Trochta et al., 2020), and these years have the most comprehensive data coverage.

#### 2.1.2 Data acquisition and indicator time‐series

We assembled data that included spatial and temporal variation in 4VWX herring across different life stages, the exploitation dynamics, and the biological and environmental factors that may influence herring. Data sources related to changes in the mean states, seasonal dynamics (timing, amplitude), and community composition of the plankton or larval assemblages that herring interact with, the type and intensity of fishing pressure, predation and competition were obtained or calculated from the stock assessments, peer‐reviewed publications, at‐sea surveys, ships of opportunity, and remote sensing are listed in Table 1. From these data, 102 indicator time‐series consisting of annual observations that could be averaged over a spatial domain (e.g., Lurcher Shoal SST) or from a single site (e.g., St. Andrew's SST), were initially developed. However, series missing >35% of their values (i.e., more than ten annual data points) between 1975 and 2005 or highly collinear with other time‐series were removed, yielding 52 time‐series (Table 2). Five of the series describe the temporal variability in larval, juvenile, and adult herring dynamics; the remaining 47 describe the environmental, biological, and anthropogenic factors that could drive them. A full description of the data sources and methods to calculate these indices are in the Supporting Information. The indices are organized within eight categories (Table 2): (1) Intrinsic (Herring; *n* = 5); (2) Anthropogenic (*n* = 5); (3) Competition (*n* = 1); (4) Predation (*n* = 6); (5) Plankton (*n* = 4); (6) Physical (*n* = 22); (7) Prey (*n* = 2); and (8) Phenology (*n* = 7).

**TABLE 1:** Data sources used to create the indicator time‐series listed in Table 2. The SI contains a detailed description of the data sources

Category	Metric	Units	Data Source	Organization	Years	Temporal	Spatial
Anthropogenic	Herring landings	Mt	CSAS assessment	DFO	1963–2016	Annual	Synoptic
Anthropogenic	Herring spatial landings	Mt	CSAS assessment	DFO	1967–2016	Annual	Synoptic
Fish	Biomass	kg m^−3^	DFO Summer RV survey	DFO	1970–2016	Seasonal	Synoptic
Fish	Abundance	N m^−3^	DFO Summer RV survey	DFO	1970–2016	Seasonal	Synoptic
Fish	Length	cm	DFO Summer RV survey	DFO	1970–2016	Seasonal	Synoptic
Fish	Herring SSB	Mt	CSAS assessment	DFO	1965–2016	Annual	Synoptic
Fish	Haddock catch‐at‐age	Mt	CSAS assessment	DFO	1970–2003	Annual	Synoptic
Larvae	Abundance	N m^−3^	Larval Herring Survey	DFO	1972–1998	Seasonal	Synoptic
Larvae	Length	µm	Larval Herring Survey	DFO	1972–1998	Seasonal	Synoptic
Zooplankton	Abundance	N m^−2^	CPR	NOAA	1971–2010	Opportunistic	Synoptic
Zooplankton	Species counts	N m^−2^	CPR	SAHFOS	1957–2013	Opportunistic	Synoptic
Zooplankton	Abundance	N m^−3^	SABS Plankton surveys	DFO	1988–2014	Seasonal	Stations
Phytoplankton	Chlorophyll	mg m^−3^	Boyce et al. 2012	NA	1890–2010	Opportunistic	Synoptic
Phytoplankton	Chlorophyll	mg m^−3^	MODIS	NASA	2003–2014	Daily	Global
Phytoplankton	Chlorophyll	mg m^−3^	MERIS	ESA	2002–2012	Daily	Global
Phytoplankton	Chlorophyll	mg m^−3^	SeaWiFS	NASA	1997–2010	Daily	Global
Phytoplankton	Greenness	PCI	CPR	NOAA	1971–2010	Opportunistic	Synoptic
Phytoplankton	Abundance	Cells m^−2^	CPR	NOAA	1971–2010	Opportunistic	Synoptic
Phytoplankton	Greenness	PCI	CPR	SAHFOS	1957–2013	Opportunistic	Synoptic
Phytoplankton	Species counts	Cells m^−2^	CPR	SAHFOS	1957–2013	Opportunistic	Synoptic
Phytoplankton	Abundance	Cells m^−2^	SABS Plankton surveys	DFO	1988–2014	Seasonal	Stations
Environment	Bottom temperature	°C	DFO Summer RV survey	DFO	1970–2016	Seasonal	Synoptic
Environment	Temperature	°C	CTD	DFO	1977–2016	Monthly	Synoptic
Environment	Salinity	PSU	CTD	DFO	1977–2016	Monthly	Synoptic
Environment	Pressure	PSI	CTD	DFO	1977–2016	Monthly	Synoptic
Environment	Sea surface temperature	°C	AVHRR Pathfinder	NOAA	1981–2016	Daily	Global
Environment	Wind speed	m s	AVHRR Pathfinder	NOAA	1981–2016	Daily	Global
Environment	Nitrate	µM	Petrie et al. 1999	DFO	1925–1996	Opportunistic	Synoptic
Environment	Phosphate	µM	Petrie et al. 1999	DFO	1925–1996	Opportunistic	Synoptic
Environment	Silicate	µM	Petrie et al. 1999	DFO	1925–1996	Opportunistic	Synoptic
Environment	Sea surface temperature	°C	DFO MEDS	MEDS	1908–2016	Monthly	Stations
Environment	Gulf Stream position	km	DFO MEDS	MEDS	1973–2016	Monthly	Synoptic
Environment	Scotian Shelf stream position	km	DFO MEDS	MEDS	1973–2016	Monthly	Synoptic
Environment	North Atlantic oscillation	Anomaly	DFO MEDS	MEDS	1950–2016	Annual	Synoptic
Environment	Wind speed	m s	DFO MEDS	MEDS	1981–2012	Annual	Stations
Environment	Wind stress	N m^−2^	DFO MEDS	MEDS	1950–2016	Annual	Stations
Environment	Atlantic multidecadal oscillation	Anomaly	NOAA	NOAA	1948–2016	Annual	Synoptic
Environment	Arctic oscillation	Anomaly	NOAA	NOAA	1950–2016	Annual	Synoptic

Abbreviations: AVHRR: Advanced Very High Resolution Radiometer; CPR: Continuous Plankton Recorder; CSAS: Canadian science advisory secretariat; CTD: Conductivity Temperature Depth; DFO: Department of Fisheries and Oceans; MEDS: Marine Environmental Data Section; MERIS: Medium Resolution Imaging Spectrometer; MODIS: Moderate Resolution Imaging Spectroradiometer; NOAA: National Oceanic and Atmospheric Administration; SeaWiFS: Sea‐Viewing Wide Field‐of‐view Sensor: SABS: St. Andrews Biological Station; SSB: spawning stock biomass.

**TABLE 2:** Indicators of herring population state and the environmental, ecological, and anthropogenic factors that affect it

*N*	Category	Index	Spatial	% missing (1975–2005)	W	λ
1	Intrinsic	Her larvae density	BoF	23	0.74	−0.2
2	Intrinsic	Her larvae length	BoF	29	0.91	−1.43
3	Intrinsic	Her recruitment	NAFO	3	0.8	0.28
4	Intrinsic	Her recruitment rate	NAFO	0	0.87	0.45
5	Intrinsic	Her SSB	NAFO	0	0.91	0.3
6	Anthropogenic	Balanced exploitation	Station	0	0.99	−1
7	Anthropogenic	Her exploitation rate	NAFO	0	0.94	0.1
8	Anthropogenic	Her landings	NAFO	0	0.95	0.15
9	Anthropogenic	Her landings 1pct	NAFO	0	0.98	0.72
10	Anthropogenic	Her landings spatial richness	NAFO	0	0.92	2.15
11	Competition	Jellyfish larvae	BoF	23	0.9	1
12	Predation	Egg predation	NAFO	6	0.95	0.35
13	Predation	Cod	NAFO	0	0.87	0.32
14	Predation	Dogfish	NAFO	0	0.9	0.38
15	Predation	Pollock	NAFO	0	0.78	0.05
16	Predation	Silver hake	NAFO	0	0.56	−0.05
17	Predation	White hake	NAFO	0	0.84	−0.05
18	Prey	Her prey	BoF	23	0.75	0.25
19	Prey	Total larval richness	BoF	32	0.94	−1.82
20	Phenology	Phyto Tpeak fall	Station	10	0.94	4.03
21	Phenology	SS current Tpeak	Basin	6	0.97	−10
22	Phenology	SST Tpeak	Pathfinder	29	0.93	9.82
23	Phenology	Stratification Tpeak	CTD	29	0.88	5.88
24	Phenology	Temperature 50m Tpeak	CTD	23	0.94	−0.88
25	Phenology	Wind Tpeak	Station	0	0.98	2.6
26	Phenology	Wind Tpeak (Pathfinder)	Pathfinder	29	0.9	−1.88
27	Plankton	Phyto (INSITU)	Phyto	35	0.93	−1
28	Plankton	Phyto state	Station	35	0.97	1
29	Plankton	Phyto diversity state	Station	32	0.97	1
30	Plankton	Phyto evenness state	Station	32	0.98	1
31	Physical	AMO	Basin	0	0.97	1
32	Physical	AO	Basin	0	0.99	1
33	Physical	GS distance	Basin	0	0.87	−2.83
34	Physical	NAO	Basin	0	0.98	1
35	Physical	Nutrients state	Station	10	0.97	1
36	Physical	Sea level	Basin	0	0.97	1
37	Physical	SS distance	Basin	0	0.97	−1.05
38	Physical	SST (CTD)	CTD	23	0.98	−1.57
39	Physical	SST duration above 12	Pathfinder	29	0.95	1.07
40	Physical	SST fall max	Pathfinder	29	0.98	−0.7
41	Physical	SST Georges Bank	Station	0	0.97	2.48
42	Physical	SST Lurcher	Station	10	0.99	0.48
43	Physical	SST Prince	Station	0	0.99	1.9
44	Physical	SST St Andrews	Station	3	0.98	−1.3
45	Physical	Temperature 50m	CTD	6	0.99	−0.28
46	Physical	Wind percent days above 10	Station	0	0.99	0.98
47	Physical	Wind speed	Station	23	0.57	4.33
48	Physical	Wind stress fall	Station	0	0.98	1
49	Physical	SST amplitude	Pathfinder	29	0.93	4.08
50	Physical	SST fall min	Pathfinder	29	0.95	−2.6
51	Physical	Wind amplitude	Pathfinder	29	0.94	−0.25
52	Physical	Wind fall max	Pathfinder	29	0.99	0.48

Abbreviations: AMO, Atlantic multidecadal oscillation; AO, Arctic oscillation; GS, Gulf Stream; NAO, North Atlantic oscillation; SS, Shelf‐Slope Water front; W=test statistic for the Shapiro‐Wilk test for normality; λ=lambda parameter for Tukey normality transformation.

Since independent data sources were unavailable, three of the five intrinsic series (spawning stock biomass; SSB, recruitment; r, and recruitment rate) combine observations (e.g. industry and DFO acoustic surveys) with an assessment model. The SSB series was calculated from calibrated VPA and acoustic estimates using methods described in Boyce et al. (2019). The recruitment series (abundance at age 1) were derived from the VPA‐based 4VWX stock assessments (Power et al., 2006; Singh et al., 2015). The recruitment rate was calculated using the approach of Platt et al. (2003) as the recruitment is standardized by SSB 3 years prior.

#### 2.1.3 Missing values, normalization, and standardization of indicator time‐series

Several time‐series contained missing values that would preclude the use of some statistical analyses, including, for example, the evaluation of time‐lagged effects. Further, case‐wise deletion would not be feasible in our analysis, as it would result in a database with <5 years of observations. Accordingly, missing values for the 52 time‐series (Table 2) were estimated using multiple imputations by chained equations (MICE; *e*.*g*. van Buuren, 2012). The MICE routine calculates the missing values and standard errors in each series based on its relationship with other series through an iterative process. An ensemble of imputations was estimated for each value, and these were used to produce accurate standard errors for the imputations. Data imputation is an increasingly common and recognized approach in marine ecology for estimating diverse types of missing data (*e*.*g*. Comte & Olden, 2017; Dahlke et al., 2020) and has been found to yield significantly less bias in subsequent analyses than case‐wise deletion (Ellington et al., 2015). Statistical descriptions of MICE are readily available (Azur et al., 2011; Patrician, 2002; Schafer, 1999; Slade & Naylor, 2020). Simulation analysis was undertaken to verify that the MICE routine would produce valid estimates of the missing values and the effect of the imputation on our subsequent analyses (see Supporting Information for details). Of the potential 1612 observations (52 series, sampled annually for 1975–2005), 199 (12%) were imputed; these values were concentrated in the plankton time‐series.

Following imputation, the time‐series were normalized if necessary, using Tukey's ladder of powers (Tukey, 1977), which finds the power transformation which maximizes normality as assessed by Shapiro–Wilkinson tests. Following the transformation, all indices were standardized to standard deviations (σ) units from the mean (Z‐scores). The normalization permitted linear methods, while the standardization allowed direct comparison of trends and relationships among variables. Consequently, relationships between variables will be expressed in standard deviations of the dependent variable per standard deviation of the independent variable, abbreviated as σ σ^−1^. Details of the MICE simulations and standardization of indicator time‐series are in the SI, where each series, consisting of imputed and nonimputed points, is plotted (Figure S1).

#### 2.1.4 Statistical analyses

Understanding how the 52 indices interact to affect the temporal variability in herring productivity is complex, involving the consideration of lagged effects, collinear predictors, and numerous permutations of predictors affecting responses. Furthermore, the number of interacting factors greatly exceeds the number of years in the time‐series, making traditional model selection impossible. In addition, modeling of key variables such as SSB could involve time offsets of potential drivers, for example, exploitation and recruitment in earlier years. Accordingly, Bayesian networks and multi‐model inference were first employed as exploratory analyses to understand the importance of all predictor series in explaining variability in the five herring responses (see SI for details). Bayesian networks are an unstructured type of machine learning whereby the strength and directionality of interactions between the 52‐indicator series are determined by the data rather than *a priori* (Scutari, 2010). Multi‐model inference allows for an ensemble set of plausible statistical models to be ranked and integrated using information theory, thereby incorporating the uncertainty of the individual models (Barton, 2015; Boyce et al., 2014, 2015; Burnham & Anderson, 2002; Johnson & Omland, 2004). Since the “saturated model” would contain far more estimated parameters than data points (52 data series, each a maximum of 31 years long), we performed a separate multi‐model analysis using a resampling routine (see SI for details). The results of the network and multi‐model inference analyses were then used to inform and build a multivariate structural equation model (SEM; Grace, 2006; Wright, 1921). The SEM tested how the 52 indicator time‐series could have interacted with each other and accounted for variability in herring population dynamics during the adult, juvenile, and larval life stages (SSB, r, and recruitment rate).

Structural equation models were estimated as a network of interacting linear models within which variables can function as both predictors and responses and where relationships between unobservable (latent) processes of interest can be estimated. SEMs are valuable for distinguishing between processes that are of interest but cannot be directly measured or observed (latent constructs) from measurements that are useful but imperfect proxies for these processes (observed variables). Whereas traditional statistical models can automatically search for and evaluate correlative relationships between predictors and the response variable, by requiring *a priori* model specification and enabling multiple pathways between variables to be simultaneously tested, SEMs facilitate a more careful and deeper consideration of causation. SEMs are becoming increasingly common in ecology (*e*.*g*. Boyce et al., 2017) and can lead to a more rigorous causal inference network than can be achieved with traditional linear models or correlative approaches (Pearl, 2009). They also permit an accounting of time‐lagged effects and multi‐collinearity. Accordingly, SEMs can also yield different results than correlative statistical approaches (Pearl, 2009). We estimated the SEM models using the complete time‐series of imputed data (1975–2005) and conducted an added sensitivity check, restricting the SEM analysis to the raw data values. The results were broadly insensitive to the use of imputed versus unimputed values. Figure S2 illustrates the steps and workflow to calculate and analyze the indicator time‐series.

## 3 RESULTS

### 3.1 Temporal variability of indicators

**FIGURE 2:**
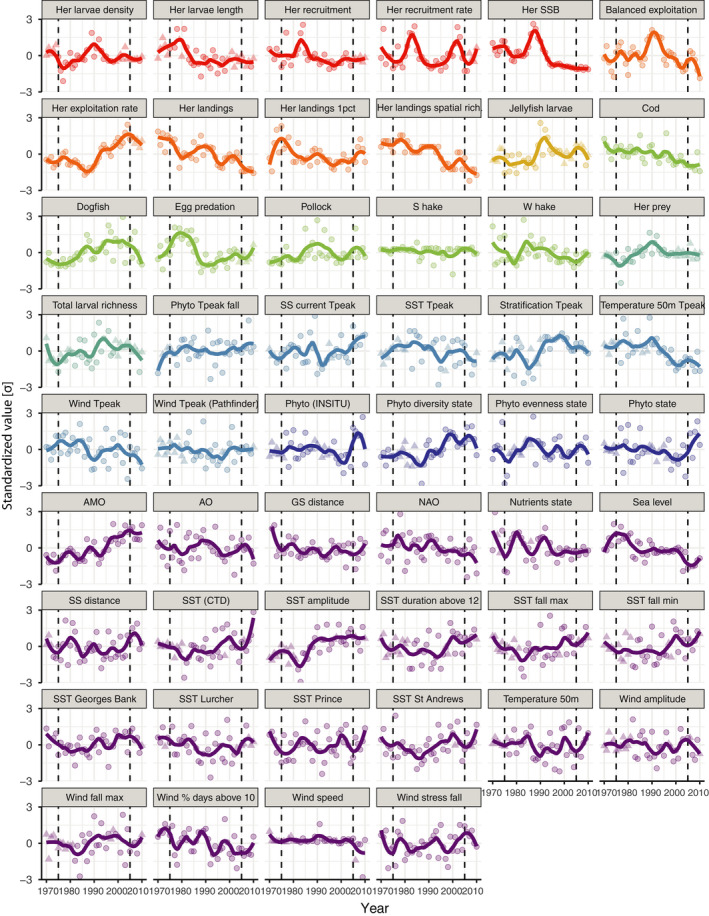
Temporal variability of imputed time‐series of all 52 environmental and biological variables used in the analyses. Standardized values are for all time‐series, and lines are interpolated with a locally estimated scatterplot smoothing (LOESS) model (span = 0.5). Circles are raw data values, and triangles are imputed. Colors depict the categories of the indices: Red is intrinsic, orange anthropogenic, yellow competition, green predation, turquoise prey, light blue phenology, dark blue plankton, and purple physical oceanographic or atmospheric. Vertical broken lines denote 1975–2005, the focus period of the statistical analysis

The 52 standardized and imputed indicator time‐series varied considerably between 1970 and 2010, featuring a broad division of variance between long and short periods (Figure 2). For example, we found that herring SSB has ~80% of its variance in periods >8 years using high‐ and low‐pass filters. In contrast, potential environmental drivers such as SST at Lurcher Shoal and the Scotian Shelf front position have ~80% in periods <8 years. Intrinsic factors related to herring population status have either declined (SSB, r, larval length) or fluctuated but remained relatively stable (larval density and recruitment rate) between 1970 and 2010 (red series in Figure 2). The intrinsic series remained stable between 2000 and 2010, except for the recruitment rate, which is highly variable. Several anthropogenic indicators suggest that exploitation on herring has significantly intensified since 1970: exploitation rate has increased sharply since ~1980, landings have declined, exploitation across age classes has become less balanced, and the geographic distribution of landings has progressively contracted (orange series in Figure 2).

The timing of seasonal development for temperature, surface wind, stratification, phytoplankton concentration, and the Scotian Shelf front position has shifted, suggesting large‐scale synchronous changes in the phenology of the environment and plankton (light blue series in Figure 2). Using linear models that accounted for temporal autocorrelation, most annual SST series had increased between 1970 and 2010, but only the one at Georges Bank had increased significantly (*p *< .05; purple series in Figure 2). The autumn SST minima and maxima, the duration of SST above 12 °C, and the seasonal amplitude across the LRA have all increased significantly (*p *< .05).

Based on all 52 standardized indicators, the largest changes between 1970 and 2010 were for the geographic distribution of herring landings (herring landings spatial richness; −0.07 σ year^−1^), the AMO (0.07 σ year^−1^), total landings (−0.06 σ year^−1^), SSB (−0.05 σ year^−1^), average larval length (−0.05 σ year^−1^), the seasonal timing of 50 m temperature (−0.05 σ year^−1^), seasonal SST amplitude (0.05 σ year^−1^), and exploitation rate (0.05 σ year^−1^), all of which were significant (*p *< .001).

### 3.2 Effects of anthropogenic, ecosystem, and climate factors on herring population production

**FIGURE 3:**
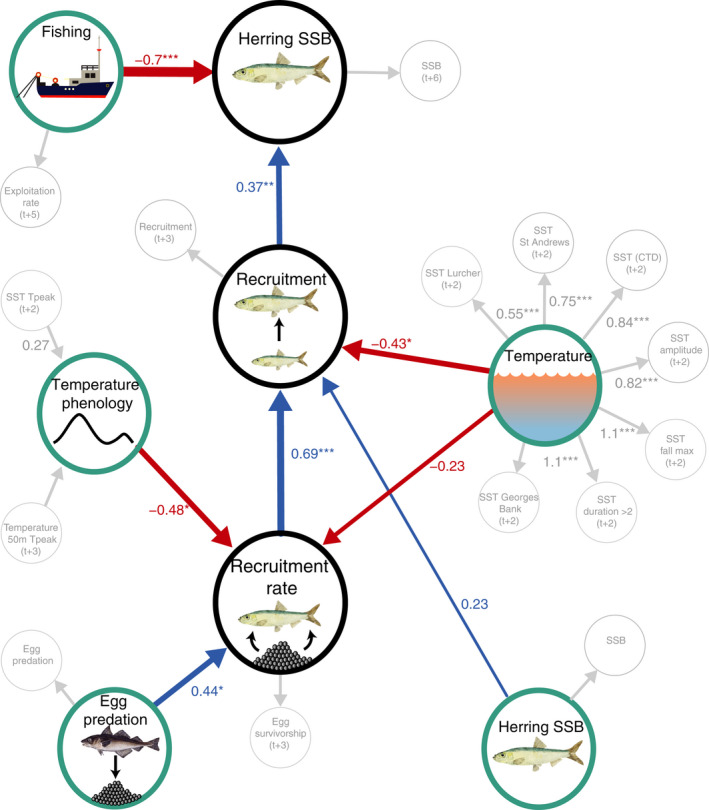
Structural equation model effects between herring population variability across different life stages and environmental and anthropogenic processes that explain it. Observed variables are depicted as smaller gray circles and are used to understand the unobservable latent processes (larger colored circles). Arrows depict the directed model relationships. Negative effects between unobservable latent processes are shown in red and positive effects in blue; the strength of these effects is in units of variance change in the response per unit variance increase in the predictor. Model effects are displayed with an asterisk denoting statistical significance (*p < .05, **p < .01, ***p < .0001). Latent processes related to intrinsic herring population status are black circles, while those related to environmental, anthropogenic, and ecological factors are turquoise circles

The direction and strength of the SEM effects were reported in standardized units of standard deviation change in one variable per unit of standard deviation change in another (σ σ^−1^). The best‐fitting SEMs accounted for 48% of the variation in herring SSB, 51% in recruitment, and 29% in recruitment rate (Figure 3).

Variability in SSB was best explained by the exploitation rate in the previous year (−0.7 σ σ^−1^; *p *< .0001) and weaker positive impacts of recruitment three years earlier (0.37 σ σ^−1^; *p *= .005; model *r* ^2^ = .48). Exploitation rate alone explained 43% of the variance in SSB, while recruitment alone explained 33%. However, an alternative SEM configuration performed equally well (*r* ^2^ = .48) but explained SSB as a positive function of the geographic distribution of fishing in the previous year (0.46 σ σ^−1^; *p *= .01) and lagged recruitment (0.57 σ σ^−1^; *p *< .0001). Under this alternative model, broader geographic distribution of fishing effort (landings) was associated with higher SSB in the following year. The geographic distribution of fishing alone explained 27% of the variance in SSB. These models fit the data equally well, emphasizing the importance of recruitment and the magnitude and geographic distribution of fishing in driving herring SSB.

Variability in herring recruitment was best explained by the rate at which spawned eggs survive to the age of recruitment (recruitment rate; 0.69 σ σ^−1^; *p *< .0001), the temperature in the previous year (−0.42 σ σ^−1^; *p *= .05), and SSB three years prior (*e*.*g*. the time interval between spawning and recruitment; 0.2 σ σ^−1^; *p *< .32; model *r* ^2^ = .51).

Lastly, variability in the recruitment rate was best explained by the index of egg predation by haddock (*Melanogrammus aeglefinus*); (0.44 σ σ^−1^; *p *= .03), temperature (−0.24 σ σ^−1^; *p *= .22), and temperature phenology (−0.48 σ σ^−1^; *p *= .03) in the previous year (*r* ^2^ = .29). However, an alternative SEM explained almost as much variability in the recruitment rate (*r* ^2^ = .28) and suggested a chain of effects initiated by haddock predation. Under this alternative model, haddock predation negatively affected larval density (−0.6 σ σ^−1^; *p *= .002), larval density in turn adversely affected larval size (−0.5 σ σ^−1^; *p *= .001), and finally, the adverse effects of temperature phenology (−0.41 σ σ^−1^; *p *= .4) and positive impact of larval size (0.4 σ σ^−1^; *p *= .01) best‐explained variability in the recruitment rate. Taken together, these two models perform equally well and emphasize the importance of both the magnitude and timing of temperature variability, as well as the role of haddock predation on the early life stages of herring.

## 4 DISCUSSION

Our analysis of 52 indicator time‐series of herring population status and environmental and biological conditions confirms the overarching importance of exploitation in driving herring population variability but also suggests that a complex interaction of anthropogenic and ecosystem drivers has contributed to the variability and long‐term decline of 4VWX herring.

**FIGURE 4:**
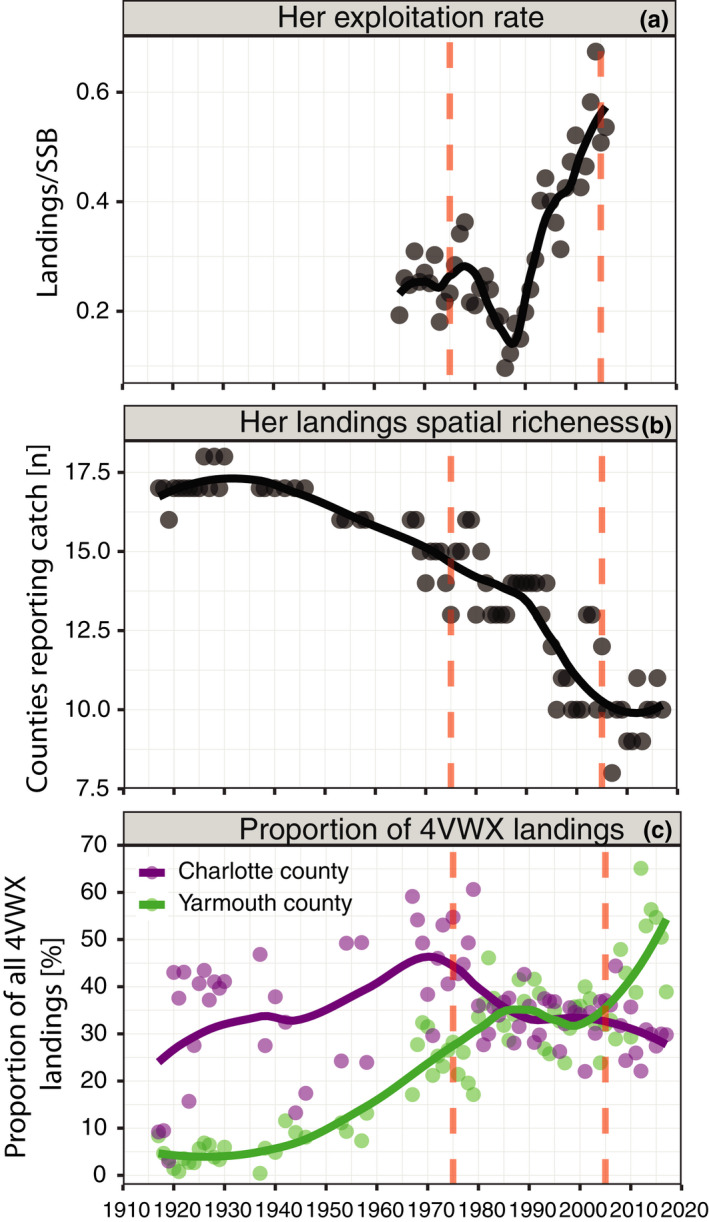
Long‐term trend in herring exploitation. (a) Exploitation rate and (b) geographic distribution of herring landings over time. (c) Change in the proportion of all 4VWX herring landings reported from Charlotte (purple) and Yarmouth (green) counties. Points are data values, and solid lines are LOESS regression lines (span = 0.5) that describe the trends; red lines depict this study's core focal period 1975–2005

Factors associated with exploitation had the most substantial and direct effect on SSB, affecting it adversely. The SSB and exploitation rate were negatively correlated (*r* = −.87); the causal SEM indicated that the exploitation rate alone accounted for 43% of SSB variance (Figure 3). Examination of the long‐term trend in exploitation rate indicated that before 1985, it had remained relatively stable and low (mean =0.25) but increased rapidly between 1986 and 2006 (from ~0.13 to 0.61, *r* = .92; mean = 0.37; Figure 4a) during a time when multiple other indicators of herring health were declining (Boyce et al., 2019). This suggests that the progressive reductions in the total allowable catch (average 132 kt, 1986–1989 to 69 kt, 2003–2006) were insufficient to prevent the long‐term decline in herring biomass and population health.

The geographic distribution of fishing was also a significant predictor of SSB, with a more geographically dispersed fishing footprint associated with higher SSB. Over the long term, the geographic footprint of herring landings has steadily declined: the number of provincial counties that reported herring landings peaked at 18 in ~1930 and declined to a minimum of 8 in 2007 (Figure 4b). The shrinking geographic distribution of fishery removals also coincides with a contraction in herring spawning locations (DFO, 2005). The progressive development of the roe fishery was possibly an aggravating factor in the declining herring status. Between the 1920s and 1970s, the majority of 4VWX herring landings were consistently reported in Charlotte County, where adjacent waters support dense aggregations of migrant juveniles (ages 1–3) from both the SWNS‐BoF and U.S. stocks (DFO, 2015). The higher proportion of Charlotte County landings before the 1980s likely reflects the early importance of juvenile herring (“sardines”; purple in Figure 4c, county time‐trends, see Figure S6). At their peak in ca. 1970, herring landings in Charlotte County accounted for 50% of the total 4VWX fishery and after that declined to their current level of 30%. Alternatively, from their minimum in ca. 1920, herring landings in Yarmouth County, primarily a roe fishery, accounted for 5% of the total 4VWX fishery, rose to about 35% in ca. 1985; after a slight decline, it currently accounts for ~55% of all landings (green in Figure 4c, Figure S6). Yarmouth County is adjacent to the 4VWX spawning locations, where herring aggregate in dense predictable concentrations and are subject to high mortality during spawning. The timing of this apparent shift from a juvenile fishery to one that primarily targets spawners for their role in the mid‐1980s also coincides with the timeline of declining herring status (Figure 1d). Taken together, these results suggest that the 1970s–1990s were a critical period for 4VWX herring: the majority of harvested herring shifted from juveniles to 4VWX prespawning adults (1970s), removals became increasingly geographically concentrated (1950s–2000s), an increasing fraction of the SSB was being harvested (>1986), and several indicators of herring population health (*e*.*g*. size, condition, metabolic state, demographic ratio) were in decline (~1960s onward; Boyce et al., 2019).

Recruitment was also a significant predictor of SSB and was, in turn, strongly affected by the survivorship during the early life stages of herring (recruitment rate; 0–3 years), with higher survivorship leading to higher recruitment. This finding broadly agrees with studies that have reported large recruitment and biomass fluctuations related to early life stage mortality and associated environmental variability (Brosset et al., 2018; Johannessen, 1980; Kotterba et al., 2017; Richardson et al., 2011). While the environmental and biological factors did not strongly affect SSB, indices related to the temperature, timing of the seasonal temperature development (phenology), and egg predation by haddock significantly influenced both the recruitment rate and absolute recruitment, which then affected adults (Figure 3).

**FIGURE 5:**
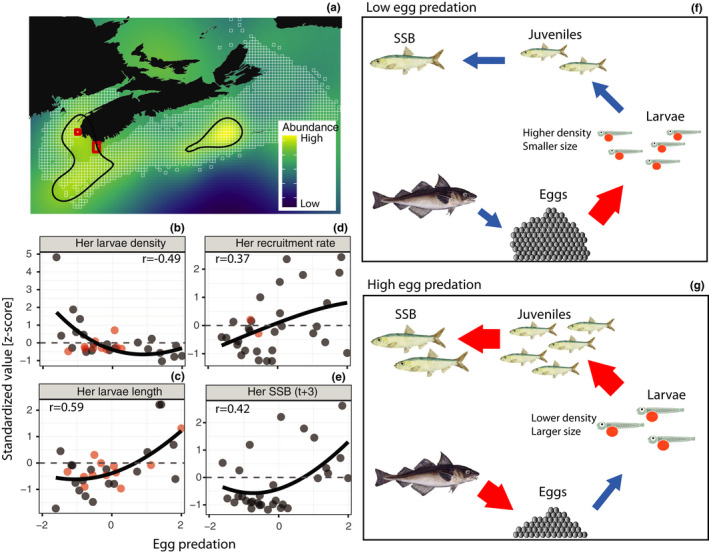
Effects of egg predation by haddock on herring population variability. (a) Long‐term average predicted abundance of haddock in July (1970–2018). Colors show predicted haddock abundances, where yellow is the highest and blue lowest abundance. White tiles depict the location of observations from which the predictions are made. Black contours show the distribution of the upper 5% of haddock abundances, and red boxes are the location of the acoustic surveys where 4VWX herring spawning occurs. Relationships between standardized egg predation intensity by haddock and (b) herring larval density (c) average larval length, (d) recruitment rate, and (e) herring SSB at *t* + 3. Lines are spline regressions (span = 3). (f) Under low egg predation, herring eggs are deposited in dense aggregations, creating density‐dependent resource limitation at the egg and larval stages, smaller eggs and larvae, and lower survivorship, recruitment, and SSB. (g) Higher egg predation reduces the density of eggs and larvae, mitigating resource limitation, facilitating larger eggs and larvae and increasing their survivorship leading to higher recruitment and SSB

The strong, consistent, and positive effects of haddock predation on the survivorship of early life stage herring (recruitment rate) were unexpected. We evaluated this relationship's robustness through sensitivity analyses and assessed numerous alternative hypotheses to explain it but could not identify any. However, several lines of evidence suggest the positive effect of haddock predation on herring may be valid. Egg mortality and predation (Johannessen, 1980), particularly by haddock (Bowman, 1922; Richardson et al., 2011; Toresen, 1991), has been emerging as an essential driver of herring population productivity in other ecosystems. Predator exclusion experiments in the Baltic Sea reported that predation accounted for 42% of herring egg mortality (Kotterba et al., 2017), while in a Norwegian fjord, Atlantic cod (*Gadus morhua*) alone were reported to consume 40–60% of spawned herring eggs (Johannessen, 1980). The importance of egg predation in our analyses is also consistent with results from a study that reported a decoupling of herring SSB and larval density on the adjacent Georges Bank due to the interaction between larval predation by haddock and exploitation pressure (Richardson et al., 2011). The geographic distribution of haddock in July further reinforces the hypothesis that they are important predators of herring eggs. Using standardized survey observations (1970–2018), we found that the areas where haddock reach peak abundances (>95th percentile) overlap substantially with the spawning areas of 4VWX herring and with Georges Bank (Figure 5A), where significant egg predation by haddock has occurred (Richardson et al., 2011).

Contrary to the findings of Richardson et al. (2011) and Kotterba et al. (2017), which reported adverse effects of egg predation on adult production, we observed a positive effect of egg predation on recruitment rate, which then propagated to recruitment and ultimately SSB lagged by three years (Figures 3 and 5). Both SEMs (Figure S5) and univariate relationships (Figure 5) suggested that the positive effect of egg predation on the recruitment rate (0.39, Figure 3) may operate by regulating the density of herring eggs (high predation →low larval density →greater larval size →higher recruitment rate). The relationships between haddock predation and larval density (*r* = −.49), larval length (*r* = .59), recruitment rate (*r* = .37), and SSB lagged by three years (*r* = .42) could affect recruitment rate *via* several pathways (Figure 5b–e). First, with all else equal, egg consumption by haddock leads to fewer eggs hatched into larvae (reduced larval density), and thus, to greater resources (planktonic prey) per individual larvae, contributing to larger larvae. Larger larvae are, in turn, better able to capture prey (Blaxter, 1962), avoid predation, and maintain position within the water column, thereby enhancing larval retention near the spawning locations where conditions are favorable (Frank, 1988; Iles & Sinclair, 1982; Stephenson et al., 2015), leading to increased survivorship. Second, herring eggs are spawned on a gravelly bottom substrate in dense aggregations that can lead to hypoxia‐driven mortality; hypoxia can also reduce hatching success by 24%–80% and lead to reduced larval sizes (DePasquale et al., 2015). Egg consumption by haddock can reduce egg density, thus reducing the extent and severity of hypoxia experienced by herring eggs, potentially increasing the hatching success of the remaining eggs and the size of subsequent larvae. Lastly, haddock egg predation can impact the deposition pattern of herring eggs, significantly affecting hatching distribution and larval size at hatching (Munk & Tosenthal, 1983). These chains of interactions from eggs to larvae, juveniles, and adults under low and high egg predation are depicted quantitatively (Figure 5b–e, and Figure S5) and schematically (Figure 5f,g).

The effect of predation on herring eggs is likely nonlinear, depending on herring eggs’ concentration and predation intensity. For example, low levels of egg predation may yield positive effects, while at some higher levels of predation, adverse effects on recruitment rate and recruitment would occur. It is possible that over the core focal period of consideration in this study (1975–2005), the magnitude of egg predation was not sufficient to induce these harmful effects. Therefore, an open and essential question is at what level the impact of egg predation would become deleterious and to what extent egg predation by other species such as adult herring themselves (Darbyson et al., 2003) may be important. We also observed an interaction between egg predation and exploitation: the largest SSB levels were predicted at the lowest exploitation rates and highest egg predation. Despite the observed effects of egg predation by haddock, the direct predation on adult herring by dogfish (*Squalus acanthias)*, pollock (*Pollachius pollachius)*, silver hake (*Merluccius bilinearis*), white hake (*Urophycis tenuis*), and Atlantic cod (*Gadus morhua*) was weak (Figure S4) and nonsignificant (Figure 3). However, due to the complex life history and many ecosystem interactions of 4VWX herring and the incomplete time‐series, the haddock egg predation index's importance may have arisen through an alternate pathway that we could not evaluate. Due to its novelty and potentially overarching impacts on herring SSB, the effect of haddock predation on early life stage herring merits further scrutiny and should be a priority for future study.

Most ocean temperature time‐series suggested warming from the 1970s throughout the area occupied by herring, particularly during autumn across the LRA. These temperature changes were negatively related to the early life stages, that is, to recruitment rate and the number of recruits but did not have a significant direct relationship with SSB (Figure 3). This finding is consistent with the reports that herring may have lower thermal tolerances when spawning and during their early life stages (larvae, juveniles) than when adults (Fassler et al., 2011; Payne et al., 2009). Due to their greater mobility and range, adult herring can also better avoid extreme warm temperatures than larvae. The more substantial temperature effects on early life stages of herring than on adults are consistent with studies that have reported narrower thermal ranges during early life stages (embryos and larvae) and reproduction (Dahlke et al., 2020; Portner & Farrell, 2008; Portner & Peck, 2010). Bioenergetics models suggest that temperature may impact herring larvae at a critical period, during yolk‐sac absorption and first feeding (Hufnagl & Peck, 2011); further, experiments suggest that such temperature increases could lead to a reduced size of newly hatched herring (Ware, 1975). Therefore, it is possible that the increasing temperatures are contributing to the reduced size of herring eggs and larvae with consequent effects on their fitness and survivorship. Indeed, warming trends over the study area support this hypothesis. Although annual average temperature trends have been moderate since 1970 (Figure 2), warming trends during the fall spawning period for herring when annual temperatures reach a maximum were stronger (Figure S7a). For example, in the LRA, the average peak SST during the herring spawning season was 13.3°C in the 1980s but increased by 3.4–16.7°C in 2012 (Figure S7b). Further, as these peak SST values are averages, observed temperatures are higher in some locations and days. Based on the reported optimal temperature for herring larvae in the eastern Atlantic derived from experiments and field studies (Moyano et al., 2020), it is likely that temperature conditions are becoming increasingly stressful to larvae and spawning adults in the LRA. The cardiac function of larval herring declines at temperatures above 16°C (Moyano et al., 2020), while growth rates have been reported to decline at temperatures above 17°C (Moyano et al., 2020) or 17.5°C (Fey, 2001). The maximum predicted temperature value for any single year and location (Figure S7a) was 19.5°C (in August of 2012 in the southwest of the LRA), a level matching the average reported upper thermal limits for adults (19.5°C) from the western Atlantic and surpassing the average of the reported upper thermal limits for spawning herring (17°C) and larvae (17°C) from the western Atlantic by 2.5°C (see Table S1). This disproportionate warming during the autumn may partly explain why the state of autumn spawning herring is declining (Boyce et al., 2019), whereas spring spawning herring in the nearby inner Bay of Fundy is not (DFO, 2015). If this warming trend continues, herring larvae and recruitment will likely become increasingly affected, and adults may soon experience direct physiological stress from these autumn temperature increases. The seasonal warming trends may also induce a shift in the phenology of autumn spawning herring or plankton development, leading to a mismatch between larval herring and their food supply (Cushing, 1990; Platt et al., 2003). In addition to temperature, the size of herring eggs can also be affected by the size and fitness of spawners (Blaxter & Hempel, 1963; Óskarsson et al., 2019; Ware, 1975), which has steadily declined since the 1980s (Boyce et al., 2019).

In undertaking this analysis, we evaluated numerous factors that could plausibly underlie the observed variability in 4VWX herring stock dynamics. Despite this, due to limited data availability and some incomplete time‐series, it was not possible to examine all variables that might reasonably play a significant role in this stock. For example, the zooplankton series that we compiled contained too many missing values to be included. It is possible that changes in the availability, timing, or quality of zooplankton prey may have affected herring dynamics, particularly during the early life stages (Brosset et al., 2018). Further, we were unable to assess the effect of some crucial predators such as whales and seabirds on herring.

Notwithstanding these caveats, this analysis emphasizes the overarching importance of exploitation as a regulator of herring productivity. However, it also highlights the importance of considering ecosystem and climate factors in the management of fisheries, particularly forage species such as herring, which traditional approaches to managing Canadian fisheries have largely overlooked (Baum & Fuller, 2016; Boyce, Fuller, et al., 2021; Boyce, Schleit, et al., 2021). Traditional approaches to managing Canadian fisheries have focused on the role of exploitation in regulating recruitment and adult biomass and often overlook the importance of ecosystem or climate factors (Baum & Fuller, 2016; Boyce, Fuller, et al., 2021; Boyce, Schleit, et al., 2021). Adapting fisheries management to include climate and ecosystem factors is a high priority for fisheries agencies worldwide and an objective within Fisheries and Oceans Canada. Despite this, analyses suggest that few fisheries in Canada are currently including these factors. A recent review reported that only 11% of 729 fisheries assessments in Atlantic Canada and the Eastern Arctic published between 2000 and 2020 mentioned climate change (Boyce, Schleit, et al., 2021).

There is a growing suite of approaches and methods for furthering the consideration of climate change in fisheries management (*e*.*g*. Boyce, Fuller, et al., 2021; Boyce, Schleit, et al., 2021; Busch et al., 2016; Gattuso et al., 2015; Holsman et al., 2019; Lawler et al., 2010; Ojea et al., 2017; Pinsky & Mantua, 2014). For example, climate vulnerability assessments (Greenan et al., 2019; Stortini et al., 2015) have been widely advocated as an approach for increasing understanding of species and fisheries vulnerability to climate changes and deploying climate adaption resources (Barange et al., 2018; Busch et al., 2016; Hare et al., 2016). Management strategy evaluations can optimize harvest rules to ensure that they are robust to future climate scenarios, population and ecosystem dynamics and other uncertainties. Dynamic management can set harvest rates based on near real‐time forecasts or respond to rapidly changing conditions (Dunn et al., 2016). The US National Oceans and Atmospheric Administration employs ecosystem models that include multiple species interactions and environmental effects to address the impact of exploitation and climate changes on the dynamics of exploited species (Holsman et al., 2017) include temperature‐dependent weight‐at‐age functions and temperature‐specific predation interactions. Integration of these approaches into scientific advice would be ideal. For example, the Alaska Eastern Bering Sea Integrated Ecosystem assessment program employs climate forecasts and projections developed by regional ocean modeling systems, food web and multispecies assessment models, and scientific surveys to support and inform fisheries decision‐making in the North Pacific (NOAA, 2021).

In addition to incorporating climate considerations, our findings also emphasize the critical importance of adopting precautionary management principles. During the 1970s and 1990s, ecosystem conditions (*e*.*g*., ocean temperature, haddock predation) were shifting, the fishery's geographic distribution was contracting, the fishery's nature was changing, the number of spawning locations was reduced, and the productivity and population health of 4VWX herring declined. These conditions served to heighten uncertainty over the herring stock's status. Overall, our results agree with recent studies that emphasize the critical importance of anthropogenic, climate (Trochta et al., 2020), and ecosystem (Kotterba et al., 2017; Richardson et al., 2011) factors in determining the early life stage dynamics and emergent adult biomass of Atlantic herring. These findings suggest that a more comprehensive ecosystem approach must be considered.

## CONFLICTS OF INTEREST

The authors declare no conflict of interest.

## AUTHOR CONTRIBUTIONS

**Daniel G. Boyce:** Conceptualization (lead); Data curation (lead); Formal analysis (lead); Investigation (lead); Methodology (lead); Visualization (lead); Writing – original draft (lead); Writing – review & editing (lead). **Brian Petrie:** Conceptualization (supporting); Data curation (supporting); Formal analysis (supporting); Funding acquisition (equal); Investigation (supporting); Methodology (supporting); Supervision (supporting); Validation (supporting); Writing – review & editing (equal). **Kenneth T. Frank:** Conceptualization (supporting); Data curation (supporting); Funding acquisition (equal); Investigation (supporting); Methodology (supporting); Validation (supporting); Writing – review & editing (equal).

## Supporting information

Supplementary Material

## Data Availability

The data used in this study are available through the Dryad digital repository (doi: https://doi.org/10.5061/dryad.gtht76hnm).
