# Supplementary material for: Fishing, predation, and temperature drive herring decline in a large marine ecosystem
Source: Ecol Evol. 2021 Dec 14;11(24):18136–50. doi: 10.1002/ece3.8411 (PMC8717267; doi:10.1002/ece3.8411)
Supplement: Supplementary file 1 — Supplementary Material [file ECE3-11-18136-s001.docx]

Supplementary Information for

**Fishing, predation, and temperature drive herring decline in a large marine ecosystem**

Daniel G. Boyce, Brian Petrie, Kenneth T. Frank

# Overview of Division 4VWX Atlantic herring stock structure

The 4VWX herring fisheries are managed as four components: *i)* Southwest Nova Scotia and Bay of Fundy, *ii)* South Shore, Eastern Shore, Cape Breton coast, *iii)* the offshore Scotian Shelf banks, and *iv)* the southwest New Brunswick migrant juveniles. Of these, the Southwest Nova Scotia and Bay of Fundy component (SWNS-BoF) dominates fisheries production, with SWNS component accounting for most of the 4WX herring landings (~93% in 2014); it has a long, detailed assessment history, with larval, juvenile, and adult herring abundance indices and associated trait data from monitoring programs using standardized protocols. Due to these factors and the uncertainties regarding components *(ii-iv)*, our analysis focuses on the SWNS-BoF herring dynamics, specifically the SWNS spawning portion. SWNS spawning herring undergo a seasonal cycle that involves separate geographic domains and differential mixing with other herring populations dependent on the life stage. Mark and recapture studies (Stobo & Fowler, 2009) suggest that adult herring (>2-3 years old) from the SWNS spawning complex undergo a cyclic seasonal migration pattern of August-November spawning near German Bank/Lurcher Shoals, January-March overwintering approximately 700 km to the northeast in Chedabucto Bay, and April-July feeding on the Scotian Shelf and Bay of Fundy. Upon hatching, the larval herring are hypothesized to be retained within an area well defined by ocean mixing regimes off SWNS (Iles & Sinclair, 1982; Stephenson, Power, Laffan, & Suthers, 2015). Juvenile herring join a spatially well-defined migrant juvenile community from other spawning complexes near Passamaquoddy Bay.

# Data and analysis workflow

The steps and workflow used to undertake these analyses are described in Figure S2.

# Data sources

## Physical and chemical

### Sea temperature and salinity

Daily 9 km^2^ sea surface temperature (SST) observations over the larval retention area were obtained from the Advanced Very High-Resolution Radiometer (AVHRR) Pathfinder Version 5.2 (PFV52) data set (Casey, Brandon, & Cornillon, 2010) between 1981 and 2016. These data were provided by GHRSST and the US National Oceanographic Data Center. The PFV5.2 data are an updated version of the Pathfinder Version 5.0 and 5.1 collections described in Casey *et al.* (2010).

Subsurface temperature and salinity profiles were obtained from shipboard samples of conductivity, temperature, and depth (CTD) vertical profiles maintained by DFO hydrographic climate databases. This database contained over 850,000 temperature-salinity profiles across the northwest Atlantic between 1920 and 2016 and was used to calculate the annual average and the timing of the seasonal peak of temperature at 50m depth.

Coastal long-term (~1930 to present) SST time-series were extracted at Halifax and St. Andrews, which were derived from weekly frontal maps and extracted from the DFO Marine Environmental Data Section (MEDS). Offshore SST series for eastern Georges Bank and Lurcher Shoal were extracted from MEDS since ~1950.

Monthly mean positions of the Gulf Stream north wall and shelf water boundary (between 50 W and 75 W) available since 1973 were extracted from MEDS.

### Stratification

An index of stratification was estimated from conductivity, temperature, and depth (CTD) vertical profiles of temperature and salinity. Seawater density (σ) was calculated from temperature and depth, and the stratification index was derived as the density differential at the surface (σ_0_) and 25 m (σ_25_), normalized to the depth difference (Behrenfeld et al., 2006; Li, 2002).

### Wind

Daily 9 km^2^ surface wind speed observations over the larval retention area were obtained from the Advanced Very High-Resolution Radiometer (AVHRR) Pathfinder Version 5.2 (PFV52) data set (Casey et al., 2010) between 1981 and 2016. These data were provided by GHRSST and the US National Oceanographic Data Center. The PFV5.2 data are an updated version of the Pathfinder Version 5.0 and 5.1 collections described in Casey *et al.* (2010).

Yarmouth winds and wind stress (1970-2016) were compiled from hourly observations made by Environment Canada and are available at their public website^[[1]](#footnote-1)^. The hourly wind velocity and wind stress data were resolved into alongshore components (along 323°), *i.e.*, forcing wind-driven ocean currents towards (323°) the Bay of Fundy or towards (143°) the Scotian Shelf. Time series of monthly averages of the wind velocity and stress was calculated from the hourly data.

### Nutrient data

Observations related to upper ocean nutrient concentrations over the region of interest were obtained from a published atlas of nitrate, silicate, and phosphate for the Scotian Shelf and Gulf of Maine (Petrie, Yeats, & Strain, 1999). The observations originate from the Canadian Marine Environmental Science Division (MESD), the National Oceanographic Data Center (NODC), universities, consulting firms, and other groups. The observations are available between 1925 and 1996 from the 0 to 500 m depth, with the number of observations increasing over time (>1960) and in shallower (<60 m) depths.

### Adjusted sea level

Monthly sea level (SL) data for Halifax and Yarmouth (1960-2013) were obtained from the Marine Environmental Data Service^[[2]](#footnote-2)^.

## Phytoplankton

### Phytoplankton abundance

Chlorophyll values (Chl; mg m^-3^) and semi-quantitative ocean colour estimates were used as proxies for marine phytoplankton biomass and derived from ship-based and remote sensing observational platforms.

### Boyce et al. 2012 Chl database

Boyce et al. (D.G. Boyce, Lewis, & Worm, 2012) compiled estimates of upper-ocean chlorophyll derived from ship-based *in situ* sampling and calibrated upper ocean transparency measurements. The database contains average upper ocean (0-20m) Chl concentrations in ocean waters >20 meters deep from 1890 to 2012. The full database, including details of the data processing, the methods used to calibrate the transparency values, and the statistical procedures used to test the accuracy and precision of the Chl values quantitatively, are in Boyce et al. 2012 (D.G. Boyce et al., 2012).

### Remote sensing Chl observations

Measurements of Chl derived from remotely-sensed ocean-leaving radiances were extracted from the National Aeronautics and Space Administration’s (NASA) ocean colour database. Chl measurements derived from the coastal zone colour scanner (CZCS; 1978-1986; Hovis et al., 1980), the Sea-viewing wide field-of-view sensor (SeaWiFS; 1997-2010; McClain, Feldman, & Hooker, 2004), the moderate-resolution imaging spectrometer (MODIS), and medium resolution imaging spectrometer (MERIS) were used. Remote sensing Chl data were extracted as either daily or weekly (8-day) averaged and at either 4 or 9 km^2^ spatial resolution.

### Continuous plankton recorder observations of ocean colour:

Semi-quantitative estimates of ocean colour derived from the continuous plankton recorder (CPR) across the Northwest Atlantic were acquired from the Canadian Department of Fisheries and Oceans (DFO) and the National Ocean and Atmospheric Administration (NOAA). The CPR is towed behind ships of opportunity at a standardized depth of 10 m, and samples are obtained as seawater flows through a 270 µm micron silk mesh within the instrument (Batten et al., 2003). To estimate phytoplankton abundance, each CPR sample is compared to a standardized colour chart and recorded on a phytoplankton colour index scale (PCI) ranging from 0 (no green) to 4 (green). Experiments have shown that these PCI categories represent a semi-logarithmic scale of increasing colour intensity such that PCI 2 samples have twice as much colour as PCI 1, and PCI 3 samples 6.5 times as much as PCI 1 (Colebrook & Robinson, 1965; Hays & Lindley, 1994). PCI estimates are available in the Northwest Atlantic since 1957. Despite the semi-quantitative nature of the PCI, studies have shown that the PCI is positively correlated to remote sensing estimates of chlorophyll (Raitsos, Reid, Lavender, Edwards, & Richardson, 2005). However, due to the large size of the silk mesh (270 µm) relative to the lower limits of the marine phytoplankton size distribution (0.2 µm), it is likely that the PCI underestimates phytoplankton biomass, particularly in oceanic waters where the average phytoplankton size is small.

### Phytoplankton species counts

A phytoplankton monitoring program has been operated by the St. Andrews Biological Station (SABS) using standardized techniques at four stations in the Bay of Fundy since 1987 (Martin, Hanke, & LeGresley, 2009). Phytoplankton species have been enumerated weekly between early May and late October, dependent on the timing of the phytoplankton bloom at Brandy Cove, Deadmans Harbour, Lime Kiln Bay, and the Wolves Islands. Samples were collected at 0, 10, 25, and 50 m depth and phytoplankton cells >5 µm were enumerated with the Utermöhl technique using a Nikon inverted microscope. While these monitoring stations are outside the main area of herring spawning, they are within a major area of aggregation for juvenile herring from the Bay of Fundy, Georges Bank, and Coastal New England.

## Zooplankton

### Continuous plankton recorder observations of zooplankton:

Estimates of zooplankton community composition and abundance at a standard depth of 10 m were derived from the continuous plankton recorder (CPR) survey (Batten et al., 2003).

The composition and abundance of plankton are assessed through 3 sequential microscopic analyses carried out on each CPR sample: i) Phytoplankton cells are identified through 20 fields of view (295 um diameter; 450X magnification), representing ~0.013% of the sample; ii) Small zooplankton individuals (<2mm) are identified through a staggered microscope traverse (2.06 mm diameter; 54X magnification), representing ~2% of the sample; and iii) Large zooplankton individuals (>2mm) are enumerated by removing them from the sample. As a trade-off between accuracy and processing speed, plankton abundances are aggregated into 12 discrete categories, such that the mean of each category is taken to be the mean for the abundance of the organism.

## Larval

### The Bay of Fundy larval herring survey

Observations of the abundance and size of larval herring were obtained from the DFO Bay of Fundy larval herring survey (Stephenson et al., 2015). Seventy-nine standardized locations were sampled (1975-1998) following international protocols (Anon, 1972). In all years, the coverage encompassed the primary herring larval area; dura­tion was ~10 days (± 7 days). We used observations available from the SWNS spawning season (September-November). Larval herring concentrations (m^-3^) were multiplied by the bottom depth to convert to larval den­sity (m^-2^).

## Fish

### DFO Ecosystem survey

Fishery-independent measurements of herring predator numbers, weights, and lengths were obtained from the Canadian Department of Fisheries and Oceans standardized summer research vessel surveys, which have been conducted annually between 1970 and 2016 across the Scotian Shelf and the Bay of Fundy. Bottom trawl surveys sample georeferenced sites annually within 24 depth-stratified strata. Multiple fishing sets were made in each stratum, with the sampling intensity proportional to stratum area. All fish species were identified and sampled for each set to provide fishery-independent data on abundances weights and lengths per tow.

# Indicator time-series

The data sources described above were used to derive the 52 indicator time-series. The approach to calculating the indicator series is described within seven categories: intrinsic, anthropogenic, physical, competition, predation, prey, plankton, and phenology. Only 3 of the 52 indicator time-series we used incorporated stock assessment model output (recruitment, SSB, and recruitment rate).

## Intrinsic

The five intrinsic indicators of herring population status (SSB, recruitment, larval density, size, and survivorship) were developed and described in Boyce *et al.* (2019) and are briefly described here.

Estimates of herring SSB and recruitment (R; age 1 abundance) were obtained from 4VWX stock assessments (Fisheries and Oceans Canada, 2015; Power et al., 2006). Since 2007, herring assessments have estimated SSB based solely on acoustic surveys conducted every two weeks on the major herring spawning during spawning (Figure S1A in Boyce *et al*. 2019) and summed to estimate the total herring SSB. The estimated, 1999-2006 linear rate of SSB decline from the VPA SSB series (-8.7 ± 1.8 t y^-1^; r^2^=0.37) was not statistically different from that of the acoustic series (-10.9 ± 5.2 t y^-1^; r^2^=0.18); (Figure S1B in Boyce *et al*. 2019). The Pearson correlation between the overlapping series was 0.75 and Generalized Least-Squares model analysis accounting for temporal autocorrelation in the response using a continuous autoregressive process indicated that the relationship was significant (p=0.03) during the overlap period (Figure S1C inset in Boyce *et al*. 2019). Based on this relationship, each series was calibrated from the other to produce a continuous time series of SSB (1965-2015; Figure S1C in Boyce *et al*. 2019). Because our analysis focuses on relative rather than absolute changes in the indices, the selection of reference series to calibrate against has no bearing.

The average annual density of larval herring was estimated from the Bay of Fundy (BoF) larval herring survey data using generalized additive models (GAMs); (Hastie & Tibshirani, 1986; Wood, 2006) as a function of year and location (see Boyce *et al*. 2019 SI for details).

The larval survivorship or r*ecruitment rate* of herring was calculated using the approach of Platt *et al.* (2003) as the number of age 1 recruits standardized by the spawning stock biomass lagged by 3 years. We selected a lag time of three years because Atlantic herring in this region are partially recruited, and three years is the age at which 75% of recruitment occurs. The annual average length of the larval herring was estimated from the BoF larval survey data using abundance-weighted GAMs (Hastie & Tibshirani, 1986; Wood, 2006) with year, location (longitude, latitude), and season (day) as predictors.

## Anthropogenic

*Balanced exploitation:* An index of the distribution of fishing removals across herring life stages. Large values denote relatively even removals across all herring life stages. The index was calculated within each year analogous to the Shannon diversity index:

$H^{'}=\sum_{i=1}^{s} -(P_{i} \times p_{i})$,

Where $H^{'}$is the index of balanced exploitation, $P_{i}$is the fraction of the total herring landings made up of age *i*, S is the numbers of age groups encountered, and ∑ is the sum from age group 1 to age group S.

*Herring exploitation rate:* the proportion of the estimated total herring biomass removed by fishing as reported in published assessments. While the magnitude of the fishing rate will strongly depend on if the herring SSB values are derived from population models (Power et al., 2006) or acoustic surveys (DFO, 2015), the relative time trend in F is largely insensitive to the approach used to derive of SSB.

*Herring landings:* Total herring removals were extracted from published stock assessment documents.

*Herring landings 1pct:* An index of how geographically concentrated herring removals from fishing were. Calculated as the proportion of all 4VWX herring landings within the provincial county that reported the highest landings. High values denote a large fraction of total removals within a single county.

*Herring landings spatial richness:* An index of how geographically distributed the herring fishery removals are. Calculated as the number of provincial counties that reported herring landings each year from the MARFIS and COMLAND landings databases.

## Physical

*AMO:* the Atlantic Multidecadal Oscillation

*AO:* Arctic Oscillation

*GS distance:* Mean position of the Gulf Stream north wall (between 50 W and 75 W) available since 1973 extracted from MEDS.

*NAO:* The North Atlantic Oscillation.

*Nutrients state:* A composite index of the upper ocean nutrient state was calculated across the LRA from a published atlas of nitrate, silicate, and phosphate for the Scotian Shelf and Gulf of Maine (Petrie et al., 1999). The observations originate from the Canadian Marine Environmental Science Division (MESD), the National Oceanographic Data Center (NODC), and universities, consulting firms, and other groups. Individual series of surface (0-20 m) nitrate, silicate, and phosphate were calculated across the LRA and standardized to units of variance (green, red & yellow in Figure S3). Once the temporal coherence of the series was confirmed, they were averaged into a composite nutrient time-series (Blue in Figure S3).

*Sea level*: The monthly sea level data described previously were added (ASL = SL + SLP) to give a time series of monthly adjusted sea levels for Halifax and Yarmouth. The difference of ASL [=ASL(Halifax) – ASL(Yarmouth)] represents the relative alongshore pressure gradient that can force currents towards or away from the Bay of Fundy.

*SS distance:* Mean position of the Scotian shelf water boundary (between 50 W and 75 W) available since 1973 extracted from MEDS.

*SST (CTD):* Annually averaged SST across the LRA calculated from CTD profiles.

*SST duration above 12:* Number of days that the daily average SST across the LRA is above 12 °C.

*SST fall max:* Maximum daily average SST over the LRA during the fall herring spawning window (July 15 – October 31).

*SST Georges Bank:* Average annual SST at the Georges Bank station extracted from the DFO Marine Environmental Data Section (MEDS).

*SST Lurcher:* Average annual SST at the Lurcher Shoal station extracted from the DFO Marine Environmental Data Section (MEDS).

*SST Prince:* Average annual SST at the Prince 5 station extracted from the DFO Marine Environmental Data Section (MEDS).

*SST St Andrews:* Average annual SST at the St. Andrews station extracted from the DFO Marine Environmental Data Section (MEDS).

*Temperature 50m:* Average annual temperature at 50m depth within the LRA calculated from CTD profiles.

*Wind percent days above 10:* The number of days where the surface wind speed is above 10 m s^-1^ across the LRA.

*Wind speed:* Average annual surface wind speed across the LRA.

*Wind stress fall:* A time-series of surface wind stress during the fall spawning window (July 15 – October 31) across the LRA was calculated from the Yarmouth winds stress (1970-2016) database described previously.

*SST amplitude:* Difference between the annual maxima and minima SST across the LRA.

*SST fall min:* Timeseries of minimum wind speed over the LRA during the fall herring spawning window (July 15 – October 31).

*Wind amplitude:* Difference between the annual maxima and minima wind speeds across the LRA.

*Wind fall max:* Timeseries of maximum wind speed over the LRA during the fall spawning window (July 15 – October 31).

## Competition

*Jellyfish larvae:* Jellyfish are likely to compete with herring for critical plankton resources. An index of jellyfish competition intensity was estimated using observations of jellyfish larvae from the BoF larval survey.

## Predation

*Egg predation:* An index of the intensity of predation on herring eggs imposed by haddock was calculated from haddock dietary ration, sizes at age, and SSB using a recently published method (Richardson, Hare, Fogarty, & Link, 2011). Using this approach, we first estimated the abundance at length of haddock using abundance-at-age and length-at-age from published Division 4WX haddock assessments (DFO, 2012; Hurley, Black, Simon, Mohn, & Comeau, 2003). Next, an individual total daily ration-at-weight was estimated by multiplying the length-weight relationship for haddock from the assessment by the daily ration as a percentage of body weight-at-weight from a published study (Daan, 1973). Finally, the annual index of haddock predation intensity was derived by summing the products of the abundance-at-length and the total daily ration-at-length. The spatial domain of the haddock assessments that we used for the calculations (4WX) overlaps significantly with that of herring (4VWX).

*Cod:* Calculated as the average number of cod per unit area within division 4X from the DFO July ecosystem survey described previously.

*Dogfish:* Calculated as the average number of dogfish per unit area within division 4X from the DFO July ecosystem survey described previously.

*Pollock:* Calculated as the average number of pollock per unit area within division 4X from the DFO July ecosystem survey described previously.

*Silver hake:* Calculated as the average number of silver hake per unit area within division 4X from the DFO July ecosystem survey described previously.

*White hake:* Calculated as the average number of white hake per unit area within division 4X from the DFO July ecosystem survey described previously.

## Prey

*Herr prey:* An index of the abundance of preferred prey of herring as reported in (Stevenson & Scott, 2005) and studies cited therein. Calculated as a time-series of the abundance of preferred prey species from the Bay of Fundy Larval herring survey across the LRA.

*Total larval richness:* Calculated as a time-series of the total number of larval species from the Bay of Fundy Larval herring survey across the LRA.

## Plankton

*Phyto (INSITU):* Average upper ocean phytoplankton abundance was calculated across the LRA from a published and publicly available database of upper ocean chlorophyll (D.G. Boyce, Lewis, & Worm, 2010; D.G. Boyce et al., 2012), described previously.

*Phyto state:* A composite index of the upper ocean phytoplankton abundance state was calculated across the LRA from a published and publicly available database of upper ocean chlorophyll (D.G. Boyce et al., 2010, 2012), and upper ocean chlorophyll estimates from the MERIS, MODIS, and SeaWiFS remote sensing platforms described previously. The index of the phytoplankton state was derived using the approach previously described for nutrients (Figure S3). Individual series of surface (0-20 m) chlorophyll was calculated across the LRA and standardized to units of variance. Once the temporal coherence of the series was confirmed, they were averaged into a composite time-series.

*Phyto diversity state:* A composite index of the upper ocean phytoplankton species diversity was calculated across the LRA from individual time-series of phytoplankton species diversity (Shannon diversity) derived from the CPR and St. Andrews Biological Station (SABS) phytoplankton monitoring databases described previously. The index of phytoplankton diversity was derived using the approach previously described for nutrients (Figure S3). Individual series of surface (0-20 m) phytoplankton species diversity was calculated across the LRA and standardized to units of variance. Once the temporal coherence of the series was confirmed, they were averaged into a composite time-series.

*Phyto evenness state:* A composite index of the upper ocean phytoplankton species evenness was calculated across the LRA from individual time-series of phytoplankton species evenness (Pielous evenness) derived from the CPR and St. Andrews Biological Station (SABS) phytoplankton monitoring databases described previously. The index of phytoplankton evenness was derived using the approach previously described for nutrients (Figure S3). Individual series of surface (0-20 m) phytoplankton species evenness was calculated across the LRA and standardized to units of variance. Once the temporal coherence of the series was confirmed, they were averaged into a composite time-series.

## Phenology

*Stratification Tpeak:* An time-series index of the timing of the peak seasonal stratification across the LRA was calculated as the central tendency. The stratification series was first centered, such that the average peak stratification for the entire series occurred on day 150. The central tendency of seasonal stratification was then calculated as:

|  | $CT= \frac{\sum_{1}^{365} d x_{d}}{\sum_{1}^{365} x_{d}}$, |  |
| --- | --- | --- |

where $d$ is the day of the year, and $x_{d}$ is the stratification on day $d$. This index has been used as a phenological indicator and has been shown to be sensitive to changes in the timing of the seasonal cycle (Asch, 2015; Colebrook, 1979; Edwards, Richardson, & Martin Edwards & Anthony J. Richardson, 2004). It is also appropriate in situations where the time-series are sampled at uneven intervals.

*Phyto Tpeak fall:* An index of the timing of the seasonal peak average upper ocean chlorophyll concentration during the fall spawning window (July 15 – October 31) across the LRA from a published chlorophyll database (D.G. Boyce et al., 2012), described previously. The timing of the fall phytoplankton bloom was calculated using the central tendency approach described for stratification.

*SS current Tpeak:* An index of the seasonal peak timing of the peak position of the Scotian Shelf water boundary (between 50 W and 75 W), using the data source described previously. The timing of the seasonal peak in the SS current position was calculated using the central tendency approach described for stratification.

*Temperature 50m Tpeak:* An index of the seasonal peak timing of the peak ocean temperature at 50m depth across the LRA, using the CTD data source described previously. The timing of the seasonal peak in temperature was calculated using the central tendency approach described for stratification.

*Wind Tpeak:* An index of the seasonal peak timing of the peak surface wind speed was calculated using the Yarmouth winds database, described previously. The timing of the seasonal peak in surface wind speed was calculated using the central tendency approach described for stratification.

*SST Tpeak:* An index of the timing of the maximum seasonal SST across the LRA. This was calculated from the AVHRR Pathfinder database. For each year, a generalized additive model (GAM) was used to estimate the seasonal variation in average SST, while statistically accounting for the nonlinear spatial variation in SST. The seasonal SST trend was estimated as a cyclic seasonal spline (basis dimension=7). From the predicted seasonal SST trend, at the herring spawning location (Lurcher Shoal; longitude=-66.3°W, latitude=43.3°N), the day of the seasonal maximum was calculated.

*Wind Tpeak (Pathfinder):* An index of the timing of the maximum seasonal surface wind speed across the LRA. This was calculated from the AVHRR Pathfinder database. For each year, a generalized additive model (GAM) was used to estimate the seasonal variation in average wind speed, while statistically accounting for the nonlinear spatial variation in wind speed. The seasonal wind speed trend was estimated as a cyclic seasonal spline (basis dimension=7). From the predicted seasonal wind speed trend, at the herring spawning location (Lurcher Shoal; longitude=-66.3°W, latitude=43.3°N), the day of the seasonal maximum was calculated.

# Analyses

## Imputation of missing time-series values

Several time-series contained missing values that would preclude the use of some statistical analyses, including, for example, the evaluation of time-lagged effects. Series missing >35% of their values between the primary focal period (1975-2005) or highly collinear with other time-series were removed. For the remaining series, missing values were estimated using multiple imputations by chained equations (MICE). Studies have reported that imputing missing values *via* MICE leads to significantly less bias in subsequent analyses than case-wise deletion (Ellington et al., 2015). Further, case-wise deletion would not be feasible in our analysis, as it would result in a database with <5 years of observations. MICE is an increasingly common and recognized approach for dealing with diverse types of missing data (*e.g.* continuous or binary). Using MICE, missing values are imputed based on the observed values for a given variable and the relationships observed in the data for other coincident variables. An ensemble of imputations is estimated for each value, and these are used to produce accurate standard errors for the imputations. If there is not much information in the observed data regarding the missing values, the imputations will be variable, leading to high standard errors. In contrast, if the observed data are highly predictive of the missing values, the imputations will be more consistent across imputations, resulting in smaller, but still accurate standard errors. Technical and statistical details of MICE are available in, for example, (Azur, Stuart, Frangakis, & Leaf, 2011; Patrician, 2002; Schafer, 1999; Slade & Naylor, 2020).

Simulation analysis was performed to guide the MICE routine and ensure that it would produce valid estimates of the missing values. From the database, 21 continuous time-series were extracted between 1970 and 2010. We randomly sampled each time-series at varying levels of missing-ness: 10%, 15%, 25%, and 25%. For each sampling intensity, we estimated the missing values using MICE using five different imputation methods: predictive mean matching, a random sample from observed value, random forest, linear regression of predicted values, and classification trees. We extracted the imputed values from the five most probable imputations. We retained the predictions from the most probable imputation and the average of the top 5 most probable imputations. For each simulation, we examined i) the correlation between the imputed and real values and ii) the correlation between all real time-series and the correlation between all time-series sampled and subsequently imputed. This routine was repeated 50 times to yield a total of 21000 imputed time-series. This simulation indicated that averaging the top five most probable imputations let to a better prediction of the true values relative to the single most probable imputation. Aside from random sampling from observed values that performed poorly, the imputation methods were approximately comparable. Random forests predicted the true values slightly better, particularly when averaging the top 5 most probabilistic imputations. The imputed values were better related to the true values when fewer observations were missing and when the variance of the time-series was lower, as expected. Based on these findings, we imputed missing values for time-series where <5% of the complete series was missing using a random forest approach and averaging the top five most probable imputed series. To maximize the information used in the imputation, the imputation was undertaken using all available data (all variables, all years), and variables that contained >5% missing values were then removed, and the temporal extent of the time-series was restricted to 1970-2010.

1. For each indicator of herring state (n=5), a multi-model analysis was undertaken (Burnham & Anderson, 2002). Multi-model inference allows for an ensemble set of plausible statistical models to be ranked and integrated using information theory, thereby incorporating the uncertainty of the individual models (D.G. Boyce, Frank, Worm, & Leggett, 2015; Daniel G. Boyce, Dowd, Lewis, & Worm, 2014; Burnham & Anderson, 2002; Johnson & Omland, 2004). The weighting was based on the Akaike Information Criterion (AIC), which helps optimize model fit and complexity (Burnham & Anderson, 2002). By eliminating the reliance on a single model, the robustness of the model inference is improved. Normalized multi-model weights for each ensemble model ($w_{m}$) were calculated as,

|  | $w_{m}=\frac{\exp\left( -\frac{1}{2\Delta_{m}} \right)}{\sum_{m=1}^{R} \exp\left( -\frac{1}{2\Delta_{m}} \right)}$ | _(2)_ |
| --- | --- | --- |

where $R$ represents the total number of models fit, and

$\Delta_{m}=\mathrm{AIC}_{m}-\mathrm{AIC}_{min}$.

Here $\mathrm{AIC}_{m}$is the AIC score for model$m$, and $\mathrm{AIC}_{min}$ is the minimum (top ranking) AIC score in the ensemble model set. This approach favours the model containing the largest amount of ‘information’ (Burnham & Anderson, 2002, 2004). The multi-model averaged parameter estimates are then derived as

|  | $\bar{\beta}_{pred,MM}= \sum_{m=1}^{R} w_{m}\beta_{pred,m}$, | _(3)_ |
| --- | --- | --- |

where $\bar{\beta}_{pred,MM}$ is the multi-model ensemble-averaged parameter estimate of the effect of the predictor on the response, $w_{m}$ are the model weights, and $\beta_{pred,m}$ are the parameter estimates for each model. The multi-model importance of each predictor was calculated as $\sum\Delta_{m}$ for each model containing the predictor, and therefore ranges between 0 and 1.

Of the potential 1612 observations (52 series, sampled annually for 1975-2005), 199 (12%) were imputed; these values were concentrated in the plankton time-series. However, many of the imputed SST series originated from the SST Pathfinder time-series that runs 1981-2005, for which observations cover the critical period when herring SSB declines. For the phytoplankton series, CPR and SABS observations covered most of the 1975-2005 period, yet there were gaps in the series, and the series from MODIS, MERIS and SeaWiFS began >=1997 and thus did not merit inclusion.

The MICE analyses were made in the R statistical computing platform (R Core Team, 2021) using the *mice* package (Buuren & Groothuis-Oudshoorn, 2011).

## Normalization and standardization of indicator time-series

All indices were transformed (normalized) using Tukey’s ladder of powers (Tukey, 1977), which finds the power transformation which maximizes normality as assessed by Shapiro-Wilkinson tests. Estimated parameters for the transformations ($\lambda$) were implemented as

$$f\left( x \right)= \left\{ \begin{aligned} x^{\lambda}, \lambda>0 \\ ln(x), \lambda=0 \\ -1 \times x^{\lambda}, \lambda<0 \end{aligned} \right\}$$

Where $x$ is the index to be transformed, and $\lambda$ is the estimated transform coefficient (Tukey, 1977). Following the transformations, all indices were standardized to unit variance. The normalization permitted the use of linear methods, while the standardization allowed direct comparison of trends. Some indices were inverse transformed so that positive values denote positive states.

## Bayesian Networks

Bayesian networks are an unstructured type of machine learning whereby strength and directionality of interactions between the 52 indicator series are determined by the data rather than *a priori*. Bayesian networks represent a set of variables and their conditional dependencies. All of the Bayesian network analyses were made in the R statistical computing platform (R Core Team, 2021) using the *bnlearn* package (Scutari, 2010).

## A multi-model analysis of predictor importance

To understand the importance of different factors in explaining herring dynamics while holding the effect of other effects fixed, we undertook a series of multi-model analyses. Multi-model inference allows for an ensemble set of plausible statistical models to be ranked and integrated using information theory, thereby incorporating the uncertainty of the individual models (D.G. Boyce et al., 2015; Daniel G. Boyce et al., 2014; Burnham & Anderson, 2002; Johnson & Omland, 2004). Since the ‘saturated model’ would contain far more estimated parameters than data points (52 data series each a maximum of 31 years long), we performed a separate multi-model analysis using a resampling routine. For each of 5000 iterations, ten predictors were randomly sampled from the set of up to 49 predictors, and all possible model permutations containing different combinations of these ten predictors were fitted. The multi-model importance of each of 10 predictors was calculated and saved. This procedure was repeated 5000 times to test all possible permutations by which the predictors could explain herring temporal variability. We began by using the multi-model routine to explore factors that drive adult SSB, and let the results dictate which early life stage indices would be most important; the multi-model analysis was then repeated to understand the effect of the predictors in explaining variability in these indices. Based on this analysis, herring SSB, recruitment, and larval survivorship rate were identified as the strongest indicators of herring population status across life stages (Figure S4). Only predictors that could directly affect the responses were considered, and lagged effects were also used where necessary. The multi-model inference analyses were made in the R statistical computing platform (R Core Team, 2021)using the *bnlearn* package (Barton, 2015).

## Multivariate structural equation model

We constructed a multivariate structural equation model (SEM) (Grace, 2006; Wright, 1921) to understand how intrinsic herring population processes relate together and how additional interrelated factors drive them. SEMs are estimated as a network of interacting linear models within which variables can function as both predictors and/or responses and within which relationships between unobservable (latent) processes of interest can be estimated. SEMs are valuable tools for distinguishing between processes that are of interest but cannot be directly measured or observed (latent constructs) from measurements that are useful but imperfect proxies for these processes (observed variables). SEMs are widely used to investigate complex causal networks in social sciences and are becoming increasingly common in ecology*^e.g.^* (Duffy et al., 2015; Mora et al., 2011). SEMs can be used to develop a more rigorous and robust causal inference network than can be achieved with a traditional linear model or correlative approaches (Pearl, 2009). SEMs also enabled us to account for time-lagged effects and multi-collinearity. The number of possible SEM permutations that could be fitted to the 50 indices is immense. Our consideration of possible SEM configurations was guided by our multi-model analysis and by fitting Bayesian networks. Contrary to SEMs, Bayesian networks do not require a pre-specified model structure and are a type of machine learning whereby the strength and directionality of network effects are determined by the data, rather than the modeler. We used the Bayesian network to inform the SEMs. The structural equation model analyses were made in the R statistical computing platform (R Core Team, 2021) using the *lavaan* package (Rosseel, 2012).

## Time lags

The statistical analysis in this study required the evaluation of how the anthropogenic, environmental and ecological conditions could impact herring at different stages of their life history development. To achieve this, we developed time lags among the anthropogenic, environmental, ecological, and intrinsic herring time-series. The survivorship of eggs and larvae (recruitment rate) was calculated as the number of recruits standardized by the spawning stock biomass lagged by 3 years (t-3). A lag time of three years was selected because Atlantic herring in this region are partially recruited, and three years is the age at which 75% of recruitment occurs (DFO, 1988). The effect of herring SSB on recruitment was thus also evaluated at a lag time of 3 years (t-3). The impact of egg and larvae survivorship on SSB was evaluated by lagging SSB by an additional 3 years (t+3); three years is the age at which herring landings generally peak in the purse seine fishery (DFO, 2015). The effect of haddock predation on herring eggs on adult herring SSB was therefore evaluated at a lag time of six years (t+6). Likewise, it was assumed that any effect of the abundance and condition of herring larvae on the survivorship of eggs or larvae to recruitment would be lagged by three years (t-3) and on adult SSB by six (t-6).

## Multi-collinearity

Multi-collinearity arises when there is a high correlation among predictor variables in a multiple regression model. While multi-collinearity can affect the validity of regression parameters estimated in traditional statistical models, SEMs can accommodate multi-collinearity via latent effects that are assumed to be correlated. We evaluated the multi-collinearity in our database of indices by calculating the variance inflation factor (VIF) between subsets of indices (Fox & Monette, 1992). VIF assessed the amount of multi-collinearity in a set of multiple regression variables and is equivalent to the ratio of the total model variance to the variance of a model that includes every single predictor. Within this framework, VIF values above 5 are interpreted as having high collinearity. The VIF analysis suggested collinearity among clusters of indices that were then used to construct latent SEM effects. For instance, multi-collinearity was observed among several temperature indices (VIF range=~1-12), justifying the creation of a latent effect for temperature.

## Sensitivity analyses

We evaluated the sensitivity of the analyses to the imputation of missing data values in the time-series. The SEM models were fitted using the complete time-series that were imputed (e.g. Figure 3) and using the raw data values. The estimated SEM effects from the imputed time-series were broadly similar to those of the raw data values in both direction and magnitude. The reported correlations between the egg predation index and herring larval density and average length, recruitment rate, and SSB were also calculated using imputed and raw series. Again, the results were broadly similar. The correlation between egg predation and SSB and recruitment rate was identical, while that of larval herring length changed from 0.59 to 0.58 when using raw series instead of imputed. The correlation between egg predation and larval density was larger (r=-0.57) when using the raw values relative to imputed (r=-0.48). Overall the use of imputed values did not overly change the results nor our interpretation of them.

## References

Anon. (1972). Working group on joint survey of larval herring in Georges bank/Gulf of Maine areas (ICNAF Subareas 4X, 5Y, 5Z, 9112 may 1972). *International Commission for the Northwest Atlantic Fisheries*, *72/123*(2852), 1–39.

Arula, T., Raid, T., Simm, M., & Ojaveer, H. (2016). Temperature-driven changes in early life-history stages influence the Gulf of Riga spring spawning herring (Clupea harengus m.) recruitment abundance. *HYDROBIOLOGIA*, *767*(1), 125–135. doi: 10.1007/s10750-015-2486-8

Asch, R. G. (2015). Climate change and decadal shifts in the phenology of larval fishes in the California Current ecosystem. *Proceedings of the National Academy of Sciences*, 201421946. doi: 10.1073/pnas.1421946112

Azur, M. J., Stuart, E. A., Frangakis, C., & Leaf, P. J. (2011). Multiple imputation by chained equations: what is it and how does it work? *International Journal of Methods in Psychiatric Research*, *20*(1), 40–49. doi: 10.1002/mpr.329

Barton, K. (2015). *MuMIn: Multi-Model Inference*. Retrieved from http://cran.r-project.org/package=MuMIn

Batten, S. D., Clark, R., Flinkman, J., Hays, G. C., John, E., John, A. W. G., … Walne, A. (2003). CPR sampling: the technical background, materials and methods, consistency and comparability. *Progress in Oceanography*, *58*(2–4), 193–215. doi: 10.1016/j.poccan.2003.08.004

Behrenfeld, M. J., O’Malley, R. T., Siegel, D. a, McClain, C. R., Sarmiento, J. L., Feldman, G. C., … Boss, E. S. (2006). Climate-driven trends in contemporary ocean productivity. *NATURE*, *444*(7120), 752–755. doi: 10.1038/nature05317

BLAXTER, J. H. S. (1960). The effect of extremes of temperature on herring larvae. *Journal of the Marine Biological Association of the United Kingdom*, *39*(3), 605–608. doi: 10.1017/S0025315400013576

Boyce, D.G., Frank, K. T., Worm, B., & Leggett, W. C. (2015). Spatial patterns and predictors of trophic control across marine ecosystems. *Ecology Letters*, *18*(10), 1001–1011.

Boyce, D.G., Lewis, M. L., & Worm, B. (2010). Global phytoplankton decline over the past century. *Nature*, *466*(7306), 591–596. doi: 10.1038/nature09268

Boyce, D.G., Lewis, M., & Worm, B. (2012). Integrating global chlorophyll data from 1890 to 2010. *Limnology and Oceanography: Methods*, *10*(11), 840–852. doi: 10.4319/lom.2012.10.840

Boyce, D.G., Petrie, B., & Frank, K. T. (2019). Multivariate determination of Atlantic herring population health in a large 1 marine ecosystem. *ICES Journal of Marine Science*. doi: 10.1093/icesjms/fsy208

Boyce, Daniel G., Dowd, M., Lewis, M. R., & Worm, B. (2014). Estimating global chlorophyll changes over the past century. *Progress in Oceanography*, *122*(January), 163–173. doi: 10.1016/j.pocean.2014.01.004

Brawn, V. M. (1960). Temperature Tolerance of Unacclimated Herring (Clupea harengus L.). *Journal of the Fisheries Research Board of Canada*, *17*(5), 721–723. doi: 10.1139/f60-057

Burnham, K. P., & Anderson, D. R. (2002). *Model Selection and Multi-Model Inference: A Practical Information-Theoretic Approach* (2nd ed.). New York: Springer-Verlag.

Burnham, K. P., & Anderson, R. P. (2004). Multimodel Inference: Understanding AIC and BIC in Model Selection. *Sociological Methods & Research*, *33*(2), 261–304. doi: 10.1177/0049124104268644

Buuren, S. van, & Groothuis-Oudshoorn, K. (2011). mice : Multivariate Imputation by Chained Equations in R. *Journal of Statistical Software*, *45*(3). doi: 10.18637/jss.v045.i03

Casey, K. S., Brandon, T. B., & Cornillon, P. (2010). The Past, Present and Future of the AVHRR Pathfinder SST Program. In *Oceanography from Space: Revisited* (pp. 1–375). doi: 10.1007/978-90-481-8681-5

Colebrook, J. M. (1979). Continuous Plankton Records: Seasonal cycles of phytoplankton and copepods in the North Atlantic ocean and the North Sea. *Marine Biology*, *51*(1), 23–32. doi: 10.1007/BF00389027

Daan, N. (1973). A quantitative analysis of the food intake of North Sea cod (Gadus morhua). *Netherlands Journal of Sea Research*, *6*(4), 479–517.

DFO. (1988). Assessment of the 1987 4WX herring fishery. *Canadian Atlantic Fisheries Scientific Advisory Committee*, *69*, 36.

DFO. (2012). Assessment of the status of 4X5Y Haddock in 2011. *Canadian Science Advisory Secretariat Sceince Advisory Report*, *023*, 1–14.

DFO. (2015). 2015 ASSESSMENT OF 4VWX HERRING Context : SUMMARY SW Nova Scotia / Bay of Fundy. *Sci. Advis. Sec. Sci. Advis. Rep.*, (July), 1–23.

Duffy, J. E., Reynolds, P. L., Boström, C., Coyer, J. A., Cusson, M., Donadi, S., … Stachowicz, J. J. (2015). Biodiversity mediates top-down control in eelgrass ecosystems: A global comparative-experimental approach. *Ecology Letters*, *18*(7), 696–705. doi: 10.1111/ele.12448

Edwards, M., Richardson, A. J., & Martin Edwards & Anthony J. Richardson. (2004). Impact of climate change on marine pelagic phenology and trophic mismatch. *Nature*, *430*(7002), 881–884. doi: 10.1038/nature02808

Ellington, E. H., Bastille-Rousseau, G., Austin, C., Landolt, K. N., Pond, B. A., Rees, E. E., … Murray, D. L. (2015). Using multiple imputation to estimate missing data in meta-regression. *Methods in Ecology and Evolution*, *6*(2), 153–163. doi: 10.1111/2041-210X.12322

Fisheries and Oceans Canada. (2015). 2015 Assessment of 4VWX Herring. *DFO Canadian Science Advisory Secretariat Advisory Report*, *2016/011*(July), 1–23.

Fox, J., & Monette, G. (1992). Generalized Collinearity Diagnostics. *Journal of the American Statistical Association*, *87*(417), 178–183.

Grace, J. B. (2006). *Structural equation modeling in natural systems*. Cambridge, UK, UK: Cambridge University Press.

Hastie, T., & Tibshirani, R. (1986). Generalized additive models. *Statistical Science*, *1*, 297–318.

Hovis, W. A., Clark, D. K., Anderson, F., Austin, R. W., Wilson, W. H., Baker, E. T., … Yentsch, C. S. (1980). Nimbus-7 coastal zone color scanner: system description and initial imagery. *Science*, *210*(4465), 60–63.

Hurley, P. C. F., Black, G. A. P., Simon, J. E., Mohn, R. K., & Comeau, P. A. (2003). Assessment of the Status of Division 4WX Haddock in 2003. *Fisheries and Oceans Canada Canadian Science Advisory Secretatiat*, *104*, 1–75.

Iles, T. D., & Sinclair, M. (1982). Atlantic Herring: Stock Discreteness and Abundance. *Science*, *215*(4533), 627–633. doi: 10.1126/science.215.4533.627

Johnson, J. B., & Omland, K. S. (2004). Model selection in ecology and evolution. *Trends in Ecology & Evolution*, *19*(2), 101–108. doi: 10.1016/j.tree.2003.10.013

Leim, A. H., Tibbo, S. N., & Day, L. R. (1957). Explorations for herring in Canadian Atlantic waters. *Bulletin of the Fisheries Research Board of Canada*, *111*, 35–83.

Li, W. K. W. (2002). Macroecological patterns of phytoplankton in the northwestern North Atlantic Ocean. *Nature*, *419*(12 September 2002), 154–157. doi: 10.1038/nature00983.1.

Martin, J. L., Hanke, A. R., & LeGresley, M. M. (2009). Long term phytoplankton monitoring, including harmful algal blooms, in the Bay of Fundy, eastern Canada. *Journal of Sea Research*, *61*(1–2), 76–83. doi: 10.1016/j.seares.2008.05.011

McClain, C. R., Feldman, G. C., & Hooker, S. B. (2004). An overview of the SeaWiFS project and strategies for producing a climate research quality global ocean bio-optical time series. *Deep-Sea Res. II*, *51*, 5–42.

Mora, C., Aburto-Oropeza, O., Ayala Bocos, A., Ayotte, P. M., Banks, S., Bauman, A. G., … Zapata, F. A. (2011). Global Human Footprint on the Linkage between Biodiversity and Ecosystem Functioning in Reef Fishes. *PLOS BIOLOGY*, *9*(4), e1000606. doi: 10.1371/journal.pbio.1000606

Moyano, M., Candebat, C., Ruhbaum, Y., Alvarez-Fernandez, S., Claireaux, G., Zambonino-Infante, J.-L. J.-L. J. L. J.-L., … Peck, M. A. (2017). Effects of warming rate, acclimation temperature and ontogeny on the critical thermal maximum of temperate marine fish larvae. *PLOS ONE*, *12*(7), 1–23. doi: 10.1371/journal.pone.0179928

Moyano, M., Illing, B., Polte, P., Kotterba, P., Zablotski, Y., Groehsler, T., … Peck, M. A. (2020). Linking individual physiological indicators to the productivity of fish populations: A case study of Atlantic herring. *ECOLOGICAL INDICATORS*, *113*. doi: 10.1016/j.ecolind.2020.106146

Patrician, P. A. (2002). Multiple imputation for missing data. *Research in Nursing & Health*, *25*(1), 76–84. doi: 10.1002/nur.10015

Pearl, J. (2009). Causal inference in statistics: An overview. *Statistics Surveys*, *3*(0), 96–146. doi: 10.1214/09-SS057

Peck, M. A., Kanstinger, P., Holste, L., & Martin, M. (2012). Thermal windows supporting survival of the earliest life stages of Baltic herring (Clupea harengus). *ICES Journal of Marine Science*, *69*(4), 529–536. doi: 10.1093/icesjms/fss038

Peck, M. A., Reglero, P., Takahashi, M., & Catalán, I. A. (2013). Life cycle ecophysiology of small pelagic fish and climate-driven changes in populations. *Progress in Oceanography*, *116*, 220–245. doi: 10.1016/j.pocean.2013.05.012

Petrie, B., Yeats, P., & Strain, P. (1999). Nitrate, silicate, and phosphate atlas for the Scotian Shelf and the Gulf of Maine. *Canadian Technical Report of Hydrography and Ocean Sciences*, *203*(Vii), 96.

Platt, T., Fuentes-Yaco, C., & Frank, K. T. (2003). Spring algal bloom and larval fish survival. *Nature*, *423*, 398–399.

Power, M. J., Clark, K. J., Fife, F. J., Knox, D., Melvin, G. D., Stephenson, R. L., … Scotia, N. (2006). 2006 Evaluation of 4VWX Herring. *DFO Canaidan Science Advisory Secretariat Research Document*, *2006/49*, 142.

R Core Team. (2021). R: A language and environment for statistical computing. In *R Foundation for Statistical Computing*. Vienna, Austria: R Foundation for Statistical Computing. Retrieved from https://www.r-project.org/

Raitsos, D. E., Reid, P. C., Lavender, S. J., Edwards, M., & Richardson, A. J. (2005). Extending the SeaWiFS chlorophyll data set back 50 years in the northeast Atlantic. *Geophysical Research Letters*, *32*, 1–4.

Reid, R. N. N., Cargnelli, L. M. M., Griesbach, S. J. J., Packer, D. B. B., Johnson, D. L. L., Zetlin, C. A. A., … Berrien, P. L. L. (1999). Essential fish habitat source document: Atlantic herring, Clupea harrengus, life history and habitat characteristics. In *NOAA Tech. Memo. NMFS-NE* (Vol. 126). Woods Hole, MA, USA.

Richardson, D. E., Hare, J. A., Fogarty, M. J., & Link, J. S. (2011). Role of egg predation by haddock in the decline of an Atlantic herring population. *Proceedings of the National Academy of Sciences*, *108*(33), 13606–13611. doi: 10.1073/pnas.1015400108

Rosseel, Y. (2012). Lavaan: An R Package for Structural Equation Modeling. *Journal Fo Statistical Software*, *48*(2), 1–36.

Schafer, J. L. (1999). Multiple imputation: a primer. *Statistical Methods in Medical Research*, *8*(1), 3–15. doi: 10.1191/096228099671525676

Scutari, M. (2010). Learning Bayesian Networks with the bnlearn R Package. *Journal of Statistical Software*, *35*(3), 1–22.

Slade, E., & Naylor, M. G. (2020). A fair comparison of tree‐based and parametric methods in multiple imputation by chained equations. *Statistics in Medicine*, *39*(8), 1156–1166. doi: 10.1002/sim.8468

Stephenson, R. L., Power, M. J., Laffan, S. W., & Suthers, I. M. (2015). Tests of larval retention in a tidally energetic environment reveal the complexity of the spatial structure in herring populations. *Fisheries Oceanography*, *24*(6), 553–570. doi: 10.1111/fog.12129

Stevenson, D. K., & Scott, M. L. (2005). *Essential Fish Habitat Source Document: Atlantic Herring, Clupea harengus, Life History and Habitat Characteristics*. Woods Hole, MA, USA.

Stobo, W. T., & Fowler, G. M. (2009). Herring Tagging in the Vicinity of the Scotian Shelf and Gulf of St. Lawrence by the Maritimes Region, 1973-1982. *DFO Canadian Technical Report of Fisheries and Aquatic Sciences 2851*, 69 p.

Tukey, J. W. (1977). *Exploratory Data Analysis*. Boston, USA: Addison-Wesley.

Wood, S. N. (2006). Generalized additive models: an introduction with R. In *Biometrics* (Vol. 62). Boca Raton,FL: Chapman & Hall/CRC. doi: 10.1111/j.1541-0420.2006.00574.x

Wright, S. S. (1921). Correlation and causation. *Journal of Agricultural Research*, *20*, 557–585.

Yin, M. C., & Blaxter, J. H. S. (1987). Temperature, salinity tolerance, and buoyancy during early development and starvation of Clyde and North Sea herring, cod, and flounder larvae. *Journal of Experimental Marine Biology and Ecology*, *107*(3), 279–290. doi: 10.1016/0022-0981(87)90044-X

## Tables and Figures

Table S1 Thermal tolerance of Atlantic herring reported in the scientific literature.

|  | **Source** | **Region** | **Upper thermal limit (°C)** |
| --- | --- | --- | --- |
| **Adults** | | | |
|  | (Brawn, 1960) | WAtl. | 19.6 |
|  | (Brawn, 1960) | WAtl. | 19 |
|  | (Brawn, 1960) | WAtl. | 19.6 |
|  | (Brawn, 1960) | WAtl. | 21.2 |
|  | (Brawn, 1960) | WAtl. | 19.5 |
|  | (Leim, Tibbo, & Day, 1957) | WAtl. | 18 |
|  | Moyano unpublished cited in (Arula, Raid, Simm, & Ojaveer, 2016) | WAtl. | 19 |
|  | (Peck, Reglero, Takahashi, & Catalán, 2013) | EAtl. | 21 |
|  | **Mean** |  | **19.6** |
|  | **Variance** |  | **1.05** |
|  |  |  |  |
| **Juveniles** | | | |
|  | (Reid et al., 1999) | WAtl. | 20 |
|  | (Peck et al., 2013) | EAtl. | 22 |
|  | **Mean** |  | **21** |
|  | **Variance** |  | **1.4** |
|  |  |  |  |
| **Larvae** | | | |
|  | (Yin & Blaxter, 1987) | EAtl. | 22.5 |
|  | (Yin & Blaxter, 1987) | EAtl. | 21.5 |
|  | (BLAXTER, 1960) | EAtl. | 23.5 |
|  | (Moyano et al., 2017) | EAtl. | 24.5 |
|  | (Yin & Blaxter, 1987) | WAtl. | 18 |
|  | (Reid et al., 1999) | WAtl. | 16 |
|  | (Peck et al., 2013) | EAtl. | 21 |
|  |  |  |  |
|  | **Mean** |  | **21** |
|  | **Variance** |  | **2.8** |
|  |  |  |  |
| **Spawning adults** | | | |
|  | (Peck, Kanstinger, Holste, & Martin, 2012) | EAtl. | 21 |
|  | (Reid et al., 1999) | WAtl. | 15 |
|  | (Peck et al., 2013) |  | 15 |
|  | **Mean** |  | **17** |
|  | **Variance** |  | **2.8** |
|  |  |  |  |
| **Eggs** | | | |
|  | (Peck et al., 2012) | EAtl. | 22 |
|  | **Mean** |  | **NA** |
|  | **Variance** |  | **NA** |

| 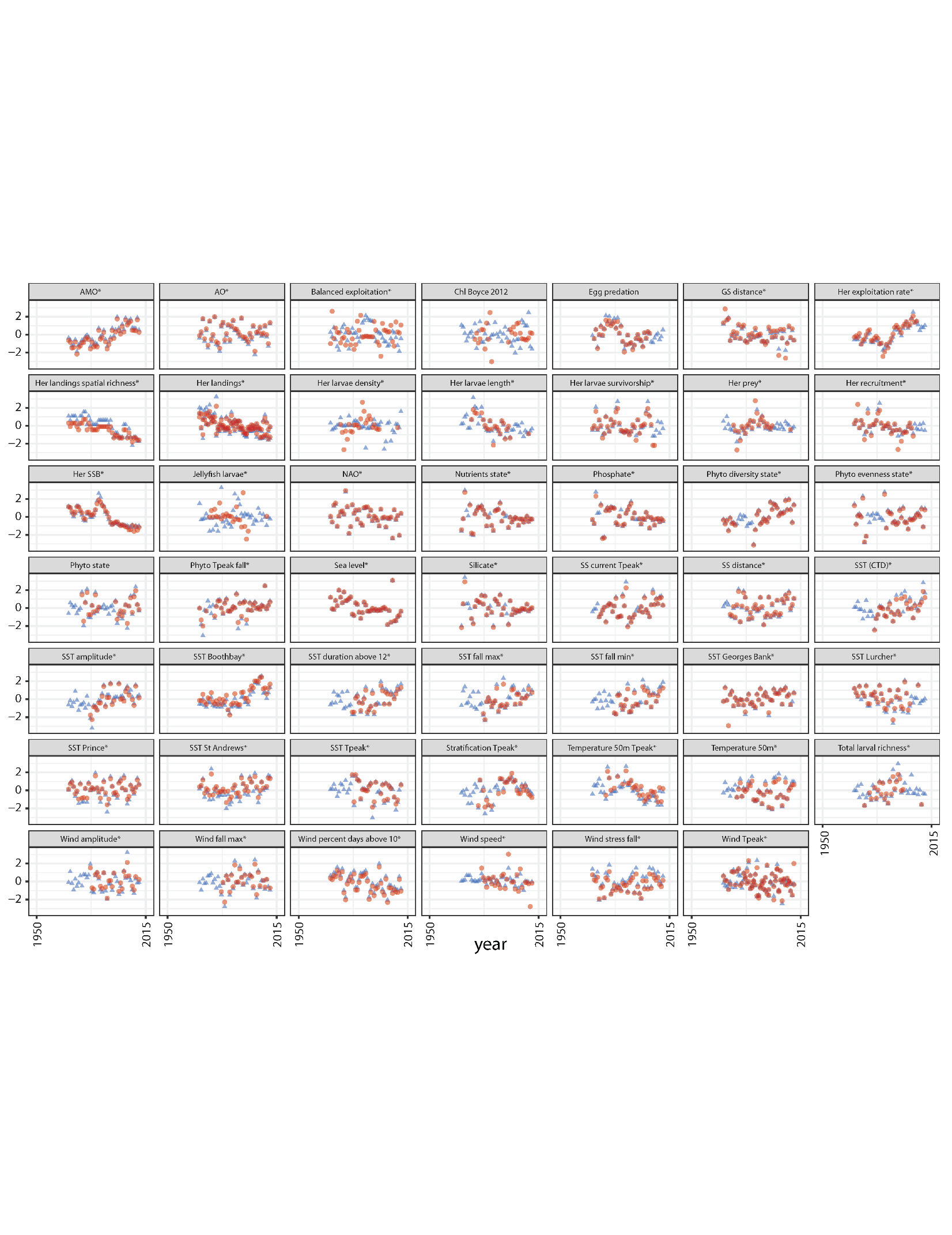  Figure S1 Raw versus imputed time-series.  Time-series of standardized indicators (red circle points) and their imputed analogues (blue triangle points) are displayed over time. Of the 52 series, 23 had no missing data, 8 had 3% to 10% missing, and 21 series had 23% to 35% missing points. |
| --- |

| 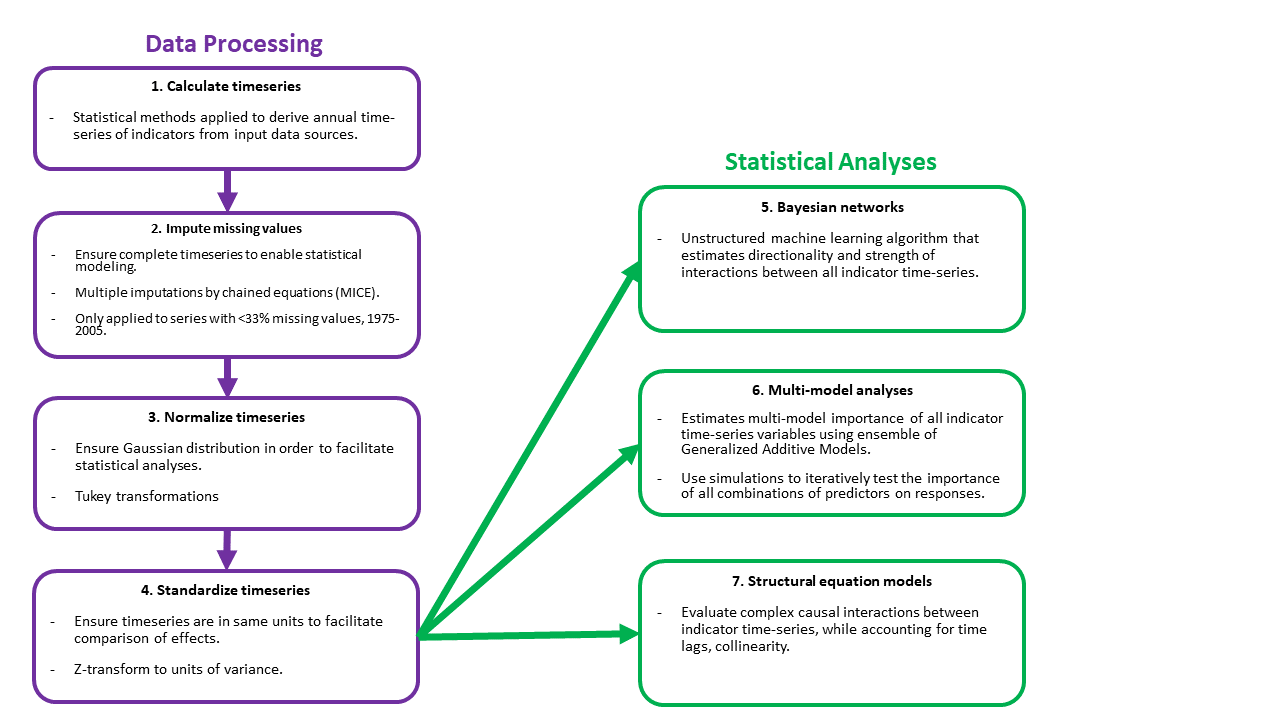  Figure S2 Workflow used in this study.  Steps involved in processing the data (purple) and the subsequent statistical analyses (green) in this study. |
| --- |

| 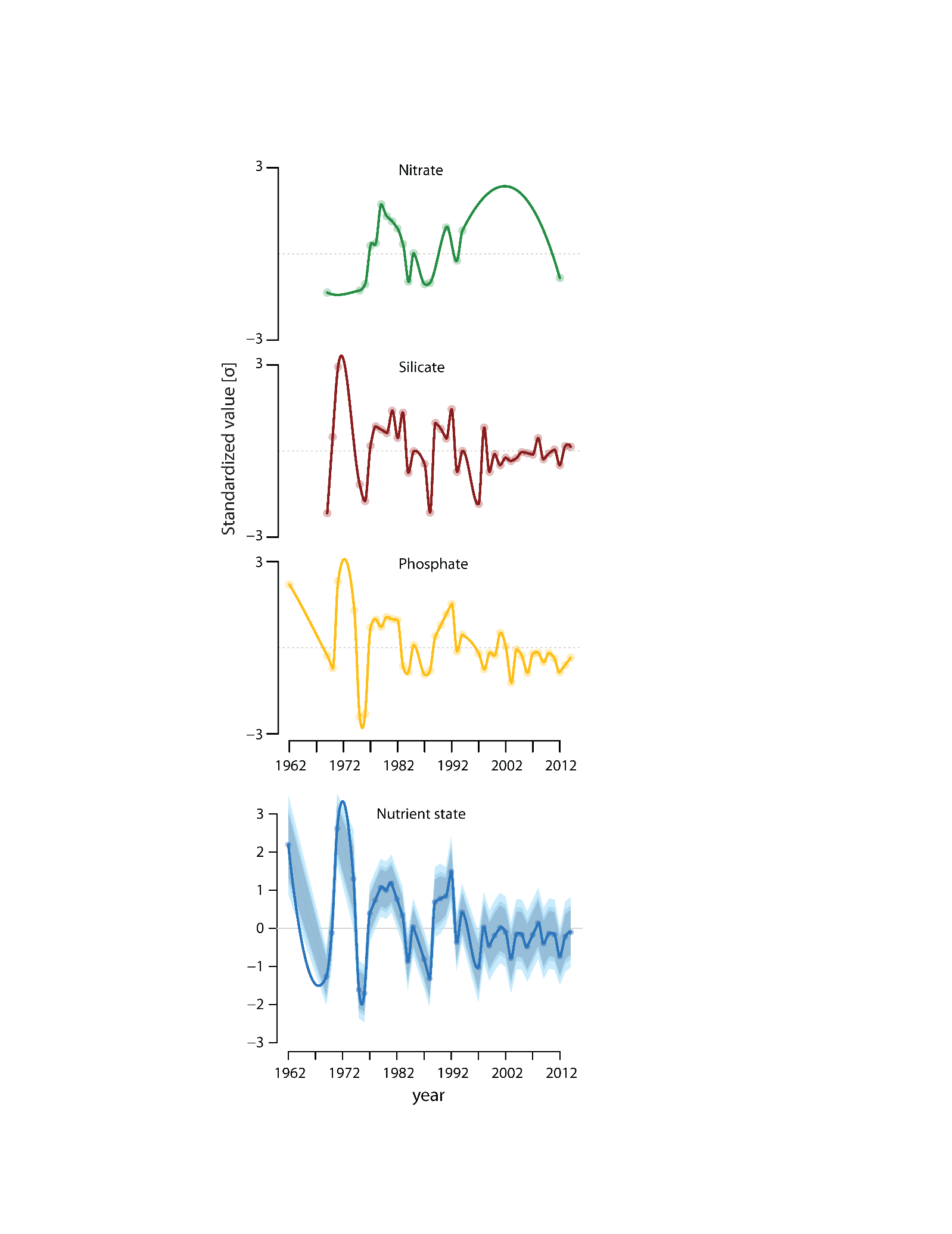  Figure S3 Derivation of nutrient state.  Time-series of average upper ocean nitrate (green), silicate (red), and phosphate (yellow) were calculated and standardized across the LRA. After confirming their strong temporal coherence, the series were averaged to create a time-series of average nutrient state (blue points and line), and its variance (shaded blue). |
| --- |

| 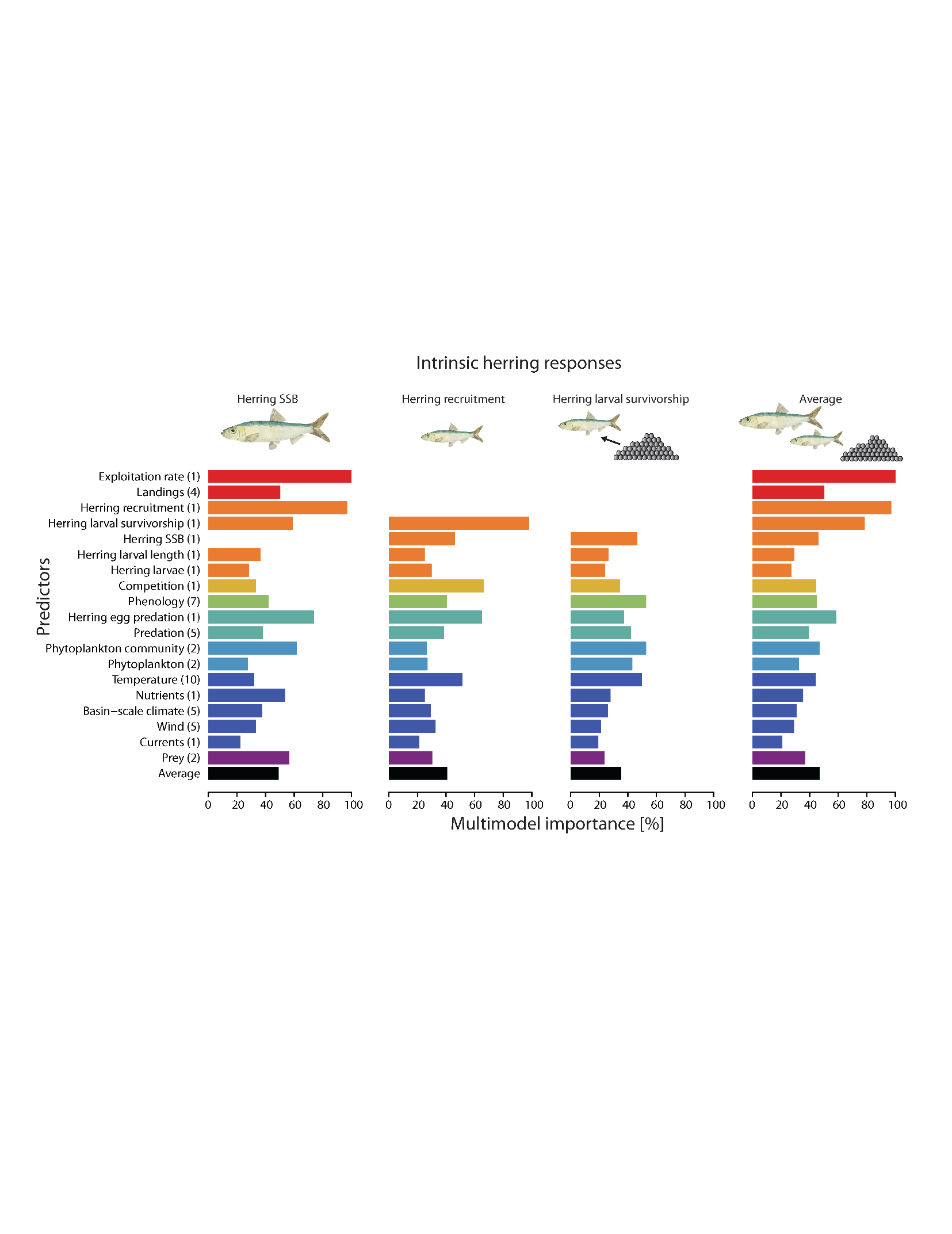  Figure S4 Multi-model importance of predictors in explaining variation in herring responses.  Colour bars represent the average standardized multi-model importance of environmental, intrinsic, and anthropogenic factors in explaining variability in herring SSB, recruitment, egg survivorship, or averaged across all stages. Multi-model importance was averaged within predictor categories (colours) and range between 0 and 100, where higher values denote greater predictor importance. The number of predictors within each category is in parentheses. |
| --- |

| 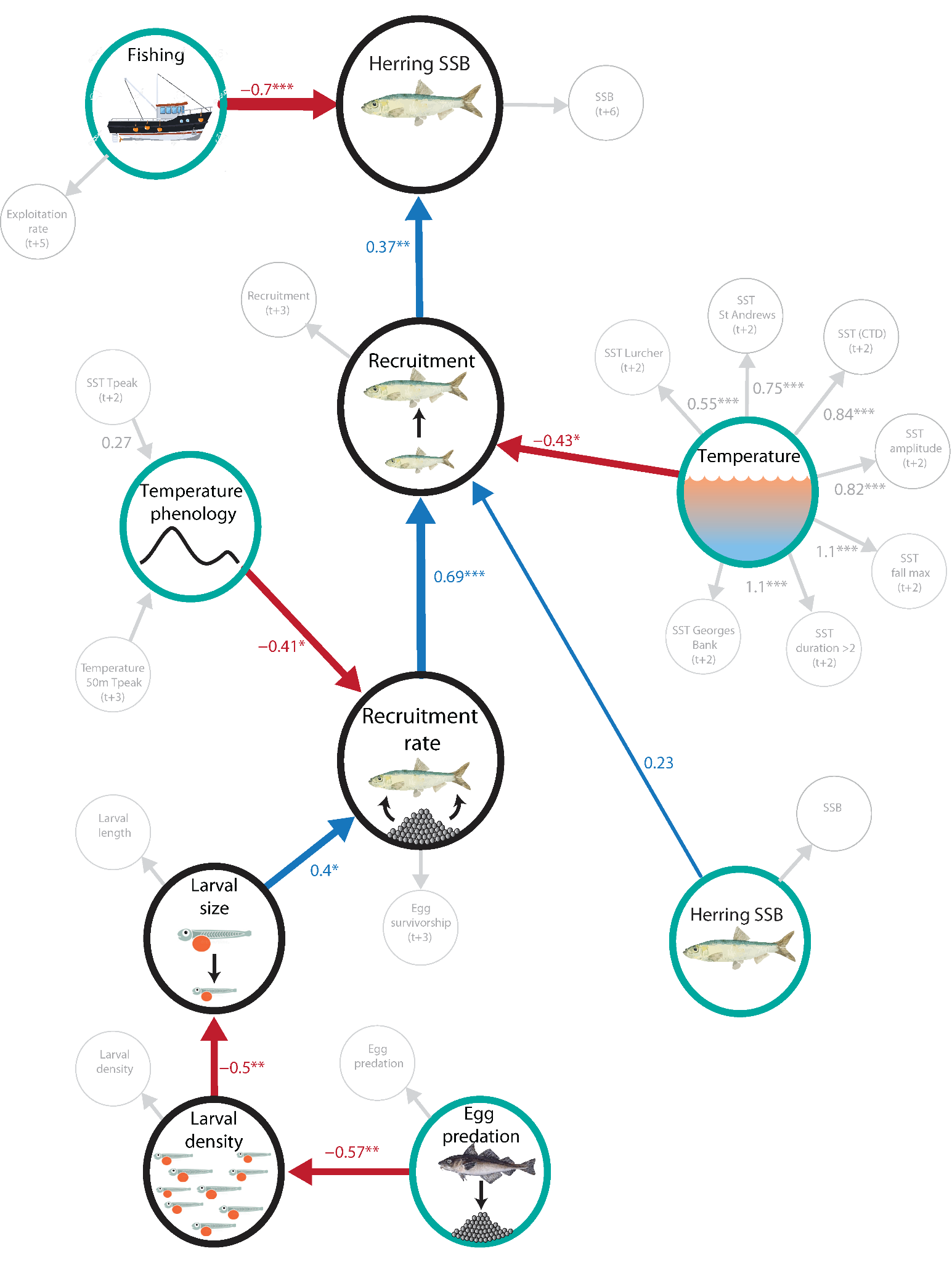  Figure S5 SEM effects between herring population variability across different life stages and processes that explain it.  Observed variables are depicted as smaller gray circles and are used to understand the unobservable latent processes (larger coloured circles). Arrows depict the directed model relationships. Negative effects are shown in blue and positive effects in red; the strength of these effects is in units of standard deviation change in the response per unit standard deviation increase in the predictor. Model effects are displayed with Asterix denoting statistical significance (*p<0.05, **p<0.01, ***p<0.0001). Latent processes related to intrinsic herring population status are red circles, while those related to environmental, anthropogenic, and ecological factors are turquoise circles. |
| --- |

| 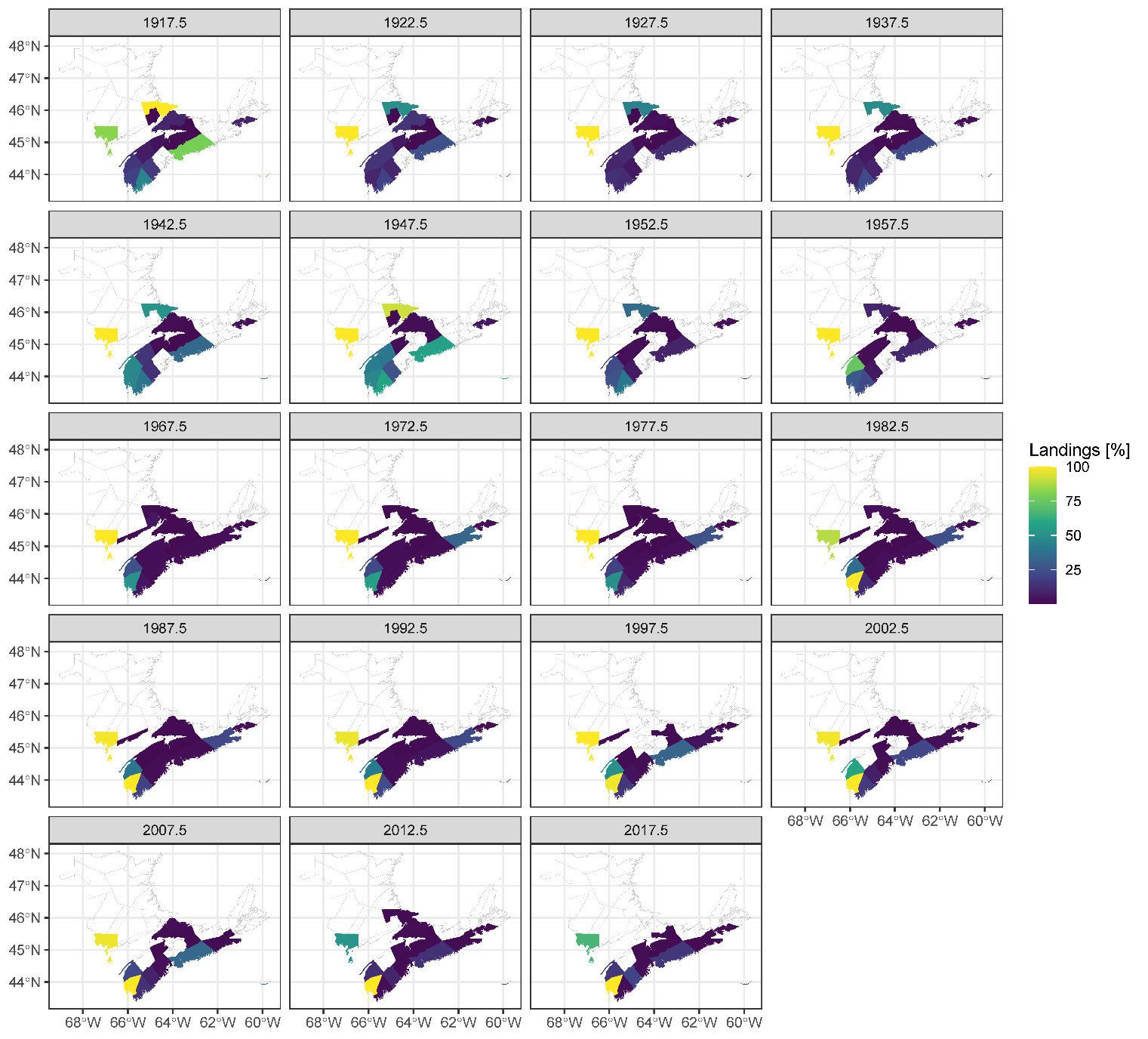  Figure S6 Changes in herring landings per county over time.  Colours depict the average herring biomass landed per municipal county within 5-year intervals. Values have been standardized within each 5-year interval as percentages of the maximum (%). Dark blue indicates lowest landings while yellow indicates greatest. Charlotte County, New Brunswick, is centred at about 45.25ᵒN, 67ᵒW; Yarmouth County, Nova Scotia, is centred about 44ᵒN, 66ᵒW. |
| --- |

| 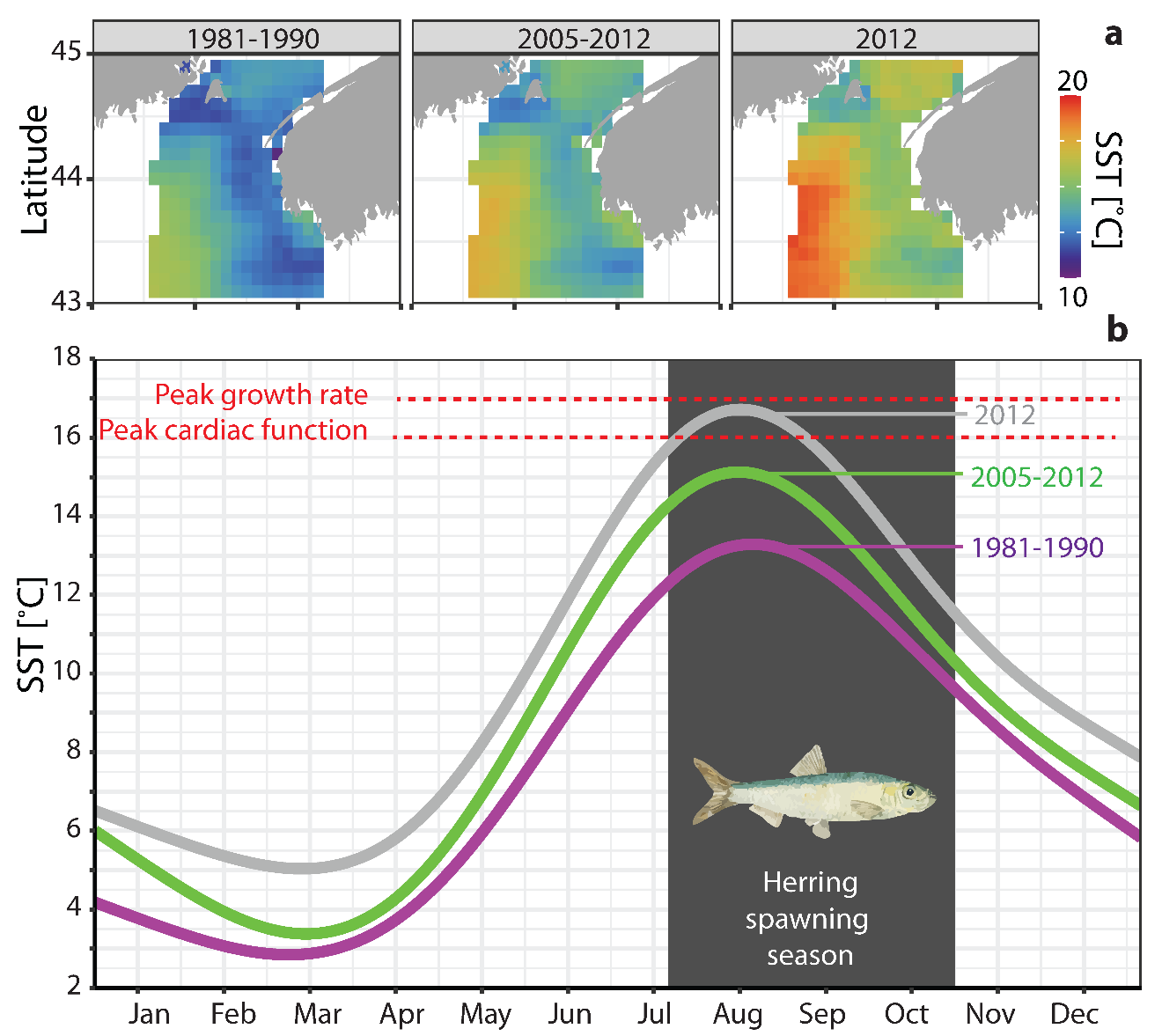  Figure S7 Effect of thermal phenology on herring population variability.  (a) Colours denote the average of the annually predicted seasonal peaks in SST at each location in the larval retention area. Annual peak SSTs were averaged between 1982 and 1990 (left), 2005 and 2012 (middle) and in 2012 (right). Red is the highest SST values, blue the lowest; white points are the primary spawning ground of German Bank (43° 20'N, 66° 24'W) and Lurcher Shoal (43° 52'N, 66° 28'W). (b) Seasonal trends in average predicted SST across the larval retention area between 1982 and 1990 (purple), 2005 and 2012 (green) and in 2012 (gray). The gray shading depicts the approximate herring spawning season, dashed red lines depict the temperatures above which peak cardiac function and growth rates for herring larvae have been reported to decline (Moyano et al., 2020). |
| --- |

1. http://climate.weather.gc.ca/historical_data/search_historic_data_e.html [↑](#footnote-ref-1)
2. http://www.meds-sdmm.dfo-mpo.gc.ca/isdm-gdsi/index-eng.html [↑](#footnote-ref-2)
